# Age‐mediated gut microbiota dysbiosis promotes the loss of dendritic cells tolerance

**DOI:** 10.1111/acel.13838

**Published:** 2023-05-09

**Authors:** Hilal Bashir, Sanpreet Singh, Raghwendra Pratap Singh, Javed N. Agrewala, Rashmi Kumar

**Affiliations:** ^1^ Immunology Laboratory CSIR‐Institute of Microbial Technology Sector 39A Chandigarh 160036 India; ^2^ Academy of Scientific and Innovative Research (AcSIR) Ghaziabad 201002 India; ^3^ Immunology Laboratory, Department of Biomedical Engineering Indian Institute of Technology, Ropar Rupnagar 140001 Punjab India

**Keywords:** aging, dendritic cells, dysbiosis, gut microbiota, immune response, tolerance

## Abstract

The old age‐related loss of immune tolerance inflicts a person with a wide range of autoimmune and inflammatory diseases. Dendritic cells (DCs) are the sentinels of the immune system that maintain immune tolerance through cytokines and regulatory T‐cells generation. Aging disturbs the microbial composition of the gut, causing immune system dysregulation. However, the *vis‐à‐vis* role of gut dysbiosis on DCs tolerance remains highly elusive. Consequently, we studied the influence of aging on gut dysbiosis and its impact on the loss of DC tolerance. We show that DCs generated from either the aged (DC^Old^) or gut‐dysbiotic young (DC^Dysbiotic^) but not young (DC^Young^) mice exhibited loss of tolerance, as evidenced by their failure to optimally induce the generation of Tregs and control the overactivation of CD4^+^ T cells. The mechanism deciphered for the loss of DC^Old^ and DC^Dysbiotic^ tolerance was chiefly through the overactivation of NF‐κB, impaired frequency of Tregs, upregulation in the level of pro‐inflammatory molecules (IL‐6, IL‐1β, TNF‐α, IL‐12, IFN‐γ), and decline in the anti‐inflammatory moieties (IL‐10, TGF‐β, IL‐4, IDO, arginase, NO, IRF‐4, IRF‐8, PDL1, BTLA4, ALDH2). Importantly, a significant decline in the frequency of the *Lactobacillus* genus was noticed in the gut. Replenishing the gut of old mice with the *Lactobacillus plantarum* reinvigorated the tolerogenic function of DCs through the rewiring of inflammatory and metabolic pathways. Thus, for the first time, we demonstrate the impact of age‐related gut dysbiosis on the loss of DC tolerance. This finding may open avenues for therapeutic intervention for treating age‐associated disorders with the *Lactobacillus plantarum*.

## Graphical abstract

Age, gut dysbiosis and loss of DC tolerance. DCs obtained from either the old or antibiotics (Abx) induced gut dysbiosis in young but not control young groups of mice exhibited loss of tolerance. This was demonstrated by their failure to optimally expand the pool of Tregs, control the overactivation of CD4^+^ T cells, upregulation of NF‐κB and pro‐inflammatory cytokines and decline in the anti‐inflammatory molecules. A significant drop in the frequency of the Lactobacillus genus is noticed in the gut. Replenishing the gut of old mice with *Lactobacillus plantarum* (LP) revived the tolerogenic function of DCs through the rewiring of inflammatory and metabolic pathways.

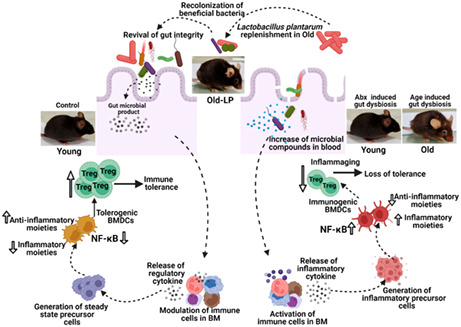

**Abbreviations**
2‐NBDG: 2‐(N‐(7‐nitrobenz‐2‐oxa‐1,3‐diazol‐4‐yl) amino)‐2‐deoxyglucose
Abx: Antibiotic
ACK: Ammonium‐chloride‐potassium bicarbonate lysis buffer
ALDH: Aldehyde dehydrogenase
ANOVA: Analysis of variance
APCs: Antigen‐presenting cells
BHI: Brain heart infusion agar
BMDC: Bone marrow‐derived dendritic cells
BTLA: B and T lymphocyte attenuator
CD: Cluster of differentiation
CFSE: Carboxyfluorescein succinimidyl ester
CFU: Colony‐forming unit
DCs: Dendritic cells
ELISA: Enzyme linked immuno sorbent assay
FBS: Fetal bovine serum
GM‐CSF: Granulocyte‐macrophage colony‐stimulating factor
HRP: Horse radish peroxidase
IDO: Indoleamine 2,3‐dioxygenase
IL: Interleukin
iNOS: Inducible nitric oxide synthase
LP: *Lactobacillus plantarum*
LPS: Lipopolysaccharide
MHC: Major histocompatibility complex
MLN: Mesenteric lymph node
NO: Nitric oxide
PDL: Programmed death‐ligand
PP: Peyer's patches
RIPA: Radioimmunoprecipitation assay
TMB: 5,5′‐Tetramethylbenzidine
Tregs: Regulatory T cells

## 1 INTRODUCTION

Age‐inflicted inflamed microenvironment favors inflammatory and autoimmune responses with a concurrent decline in the protective immunity (Kim et al., 2017). Both Innate and adaptive arms of the immune system show age‐related changes in their functional capacity, manifested by diminished antigen uptake and presentation capacity, phagocytic activity, thymic involution, antibody production, and reduced response to vaccination and infection. A gain of non‐specific innate immunity with a loss of adaptive immunity is linked with the advancement of age (Lee et al., 2022).

Dendritic cells (DCs) are professional antigen‐presenting cells (APCs) and multifaceted regulators of immunity against pathogens (Banchereau & Steinman, 1998). Furthermore, they also induce tolerance to self and innocuous antigens (Coquerelle & Moser, 2010). Recognition of pathogen's danger signals, uptake of antigens, and their processing provides developmental cues to DCs for their maturation and activation. DCs are the only APCs with the capacity to activate and differentiate naive T cells (Trombetta & Mellman, 2005). Immunogenic DCs express a higher level of MHCII and co‐stimulatory molecules CD40, CD80 and CD86, and release an elevated amount of pro‐inflammatory cytokines like IL‐12, IL‐6, IL1‐β, and TNF‐α (Hackstein & Thomson, 2004). In contrast, tolerogenic DCs display comparatively lower levels of MHCII and co‐stimulatory molecules and higher expression of inhibitory receptors such as Tim‐3 and PDL‐1 (Iberg & Hawiger, 2020). Tolerogenic DCs produce augmented quantities of anti‐inflammatory cytokines like IL‐10 and TGF‐β (Vogel et al., 2022) and reduced production of IL‐12 (Steinman et al., 2003). DCs maintain immunological tolerance by clonal deletion of T cells, induction of anergy, and generation of Tregs (Horton et al., 2017). In addition, they maintain peripheral tolerance against self‐antigens and presentation to autoreactive T cells (Hawiger et al., 2001). This heterogeneity in the functionality of DCs establishes a fine balance between the activation and suppression of the immune system. The loss of DC tolerance is a gradual process connected with aging, as observed by the increased production of pro‐inflammatory cytokines, reduced phagocytic capacity (Agrawal et al., 2017), deficient Tregs‐inducing capacity, and failure to curb autoimmune and inflammatory responses. Although enhanced activation of NF‐κB and pro‐inflammatory responses are considered responsible for this phenomenon (Agrawal et al., 2007), the mechanism connected with age‐associated DCs dysfunction remains extensively elusive.

Recently, the role of gut microbiota is increasingly being recognized in the development, maturation, and maintenance of homeostasis of the immune system through elegant experiments conducted on germ‐free mice models (Honda & Littman, 2016). Gut microbiota induces peripheral tolerance through the induction of Tregs, IgA‐secreting B cells, Th17 cells, and through the DC modulation (Zheng, Liwinski, & Elinav, 2020). An alteration in gut microbiota composition and function has been reported with age, and concomitantly, it is associated with various autoimmune diseases (Bosco & Noti, 2021). Gut dysbiosis with age results in a loss of mainly Firmicutes and Bacteroides and the predominance of Proteobacteria and therefore predisposes to increased risk of immune dysregulation and persistence of chronic inflammation (Ragonnaud & Biragyn, 2021). However, it is still largely unknown how aging provokes gut dysbiosis and loss of DC tolerance.

Taking into consideration the aforesaid facts, we studied the influence of aging on gut microbiota and its implication on the activation, differentiation, and function of DCs. Through comparison of DCs derived from young (DC^Young^), old (DC^Old^), and antibiotic‐treated young animals (DC^Dysbiotic^). We show the correlation between gut dysbiosis and loss of DC tolerance. Our study revealed that aging incited gut disruptions linked with the failure in the expansion of Tregs and downregulation of tolerance‐associated gene network and signaling pathways that resulted in the loss of DC tolerance. Importantly, the loss of DC tolerance was connected with the disappearance of the beneficial bacteria Lactobacillus. Interestingly, replenishing the gut of aged mice with *Lactobacillus plantarum*, restored the age‐associated loss of tolerance in DCs (DC^Old‐LP^). The study suggests the therapeutic role of *Lactobacillus plantarum* in the maintenance of DC tolerance and its use as a remedial measure for alleviating age‐associated immune system defects.

## 2 RESULTS

### 2.1 Bone marrow‐derived hematopoietic cells exhibit declined differentiation potential with age and gut dysbiosis to DCs

**FIGURE 1:**
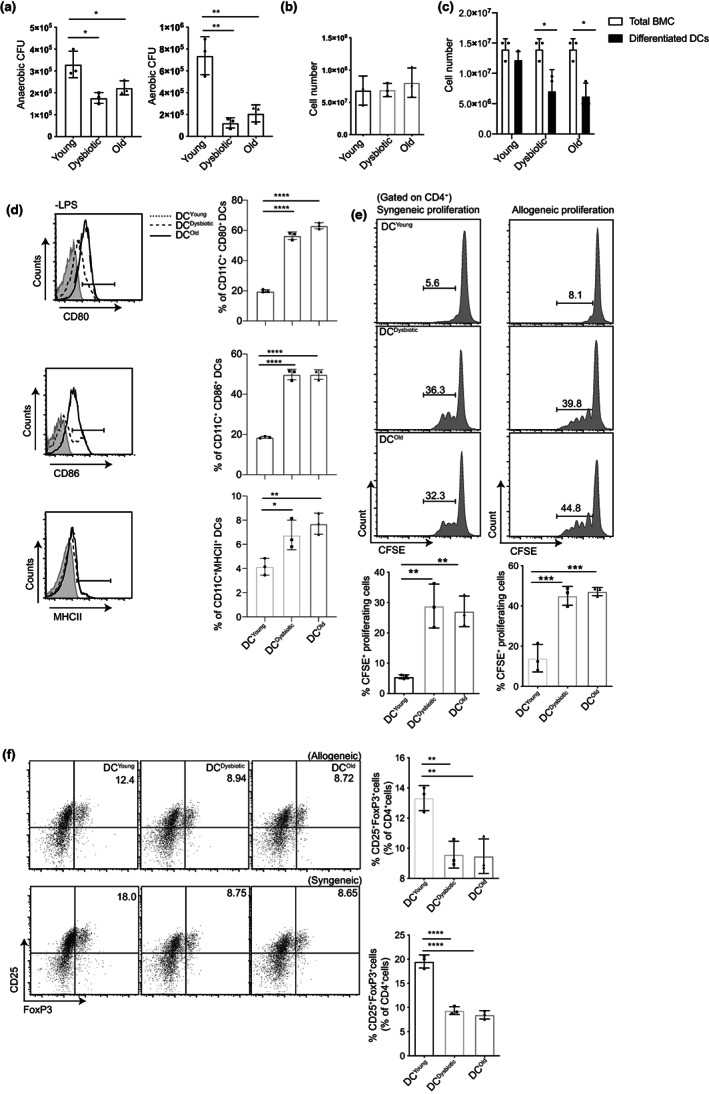
Aging and Abx treatment of young animals disrupts gut microbiota and impairs the differentiation of BMCs to DCs. Young control, young dysbiotic (21 days of antibiotics treated) and old, groups of animals were compared; (a) quantification of facultative anaerobes and aerobes bacteria from fecal matter; (b) quantification of total BMCs; (c) comparative analysis of the total number of BMCs and DCs differentiated from BMCs; (d) phenotypic (CD11c^+^CD80^+^ population (top), CD11c^+^CD86^+^(middle) and CD11c^+^MHCII^+^ (bottom) analysis of differentiated (‐LPS) DC^Young^, DC^dysbiotic^ and DC^Old^; (e) representative FACS plot and bar graph of syngeneic and allogeneic CD4^+^ T‐cell proliferation induced by DC^Young^, DC^Dysbiotic^ and DC^Old^; (f) representative FACS plot and bar graph of CD4^+^FoxP3^+^Tregs differentiation by DC^Young^, DC^Dysbiotic^ and DC^Old^. The data (mean ± SD) are from three independent experiments, with each point representing a pool of three animals for one independent experiment (*n* = 3 mice/group). Statistical analysis was done by one‐way ANOVA followed by Tukey's multiple comparison tests except (c, d), where two‐way ANOVA followed by Sidak's multiple comparisons test was done. *p < 0.05, **p < 0.01, ***p < 0.001, ****p < 0.0001.

Aged hematopoietic stem cells (HSCs) generate a dysfunctional immune system that contributes to immunosenescence (Geiger et al., 2013). Additionally, dysbiosis of the microbial population with antibiotic treatment adversely affects the bone marrow cells (Josefsdottir et al., 2017). These observations led us to evaluate the number and differentiation potential of HSCs in aged (20–22 months) and young dysbiotic mice (young mice treated with an antibiotic cocktail for 21 days) compared to young mice (2–4 months). Gut bacterial colony‐forming units (CFUs) were enumerated and compared in all three groups under anaerobic and aerobic conditions from their fecal homogenate. We observed a significant decrease in the gut bacterial load in both facultative anaerobes and aerobes in old and young dysbiotic mice in comparison with young mice (Figure 1a). Next, an absolute number of bone marrow cells (BMCs) and their differentiation potential towards dendritic cells were compared. No significant alteration was observed in the absolute cell counts among the three groups (Figure 1b) but a significant decrease in the differentiation of BMCs to DCs in both old and young dysbiotic groups (Figure 1c) compared to the young group was noticed. We conclude that gut dysbiosis, either due to old age or antibiotic treatment, impairs the differentiation potential of bone marrow/myeloid progenitor cells.

### 2.2 Age and gut dysbiosis affects the maturation and function of DCs

DCs play an important role in both, orchestrating antigen‐specific T‐cell responses and maintenance of the peripheral tolerance (Coquerelle & Moser, 2010). However, their functional capacity gets curtailed with chronological aging (Agrawal et al., 2007), and with gut microbiota modulation (Uribe‐Herranz et al., 2020). The phenotype of the DCs can ascertain their activation or tolerization function. A significant upregulation in the expression of co‐stimulatory molecules CD80, CD86, and MHCII molecules (Figure 1d), and CD40 (Figure S1c) molecules was observed on the DC^Dysbiotic^ and DC^Old^, as compared to DC^Young^ upon differentiation.

One of the mechanisms for the induction of peripheral T‐cell tolerance by dendritic cells is attributed to their capacity to phagocytose foreign pathogens, cancer cells, and self‐apoptotic cells. A defect in this capacity leads to a breach of tolerance (Savill et al., 2002). Given that we observed the mature phenotype of DCs in our experimental setup, we checked their ability for phagocytosis. We observed a significant decline in phagocytosis of the antigen (Dextran‐FITC) by both DC^Dysbiotic^ and DC^Old^ compared to DC^Young^ (Figure S1d). Additionally, we noticed a notable decrease in the engulfment of apoptotic bodies (Figure S1e). These results indicate reduced phagocytosis by DCs on aging and gut dysbiosis.

DCs induce immunogenic or tolerogenic T‐cell stimulation based on their maturation state (de Heusch et al., 2004). We next examined the effect of gut dysbiosis on the ability of DCs to activate CD4^+^ T cells. DC^Dysbiotic^ and DC^Old^ induced rigorous proliferation of syngeneic CD4^+^ T cells in comparison with DC^Young^.This was further authenticated using allogeneic CD4^+^ T cells (Figure 1e). These data suggest that DC^Dysbiotic^ and DC^Old^ exhibit hyperactivation of CD4^+^ T cells. Further to confirm this, we examined the Tregs‐inducing potential of all three DCs, as the regulatory function of DCs is well recognized for the maintenance of central and peripheral tolerance by driving naive T cells to differentiate towards Tregs (Raker et al., 2015). A significant loss of the Tregs‐inducing potential of DC^Dysbiotic^ and DC^Old^ was noted (Figure 1f). These results indicate loss of tolerogenic and acquisition of immunogenic properties in old and young dysbiotic DCs, suggesting a substantial role of gut dysbiosis in the modulation of DCs function. Next, we focused our studies on understanding the possible mechanism of action for the loss of tolerogenic effect of BMDCs with Abx treatment and old age.

### 2.3 Loss of tolerogenic potential of DCs with gut dysbiosis is primarily mediated through secreted factors and modulation of regulatory and metabolic gene expression

In both proliferation and Tregs induction experiments, total CD4^+^ T cells were stimulated through plate‐bound anti‐CD3 and soluble anti‐CD28 antibodies while co‐culturing with DCs for optimal activation of T cells. This approach nullifies the effect of phenotypic difference among DCs for the observed effect; hence, we hypothesized that gut dysbiosis with old age and antibiotic treatment instigate cell‐intrinsic properties of myeloid precursors and turn them towards immunogenic rather than tolerogenic, and this effect is mediated through secreted factors in the microenvironment of DC and T‐cell co‐culture.

**FIGURE 2:**
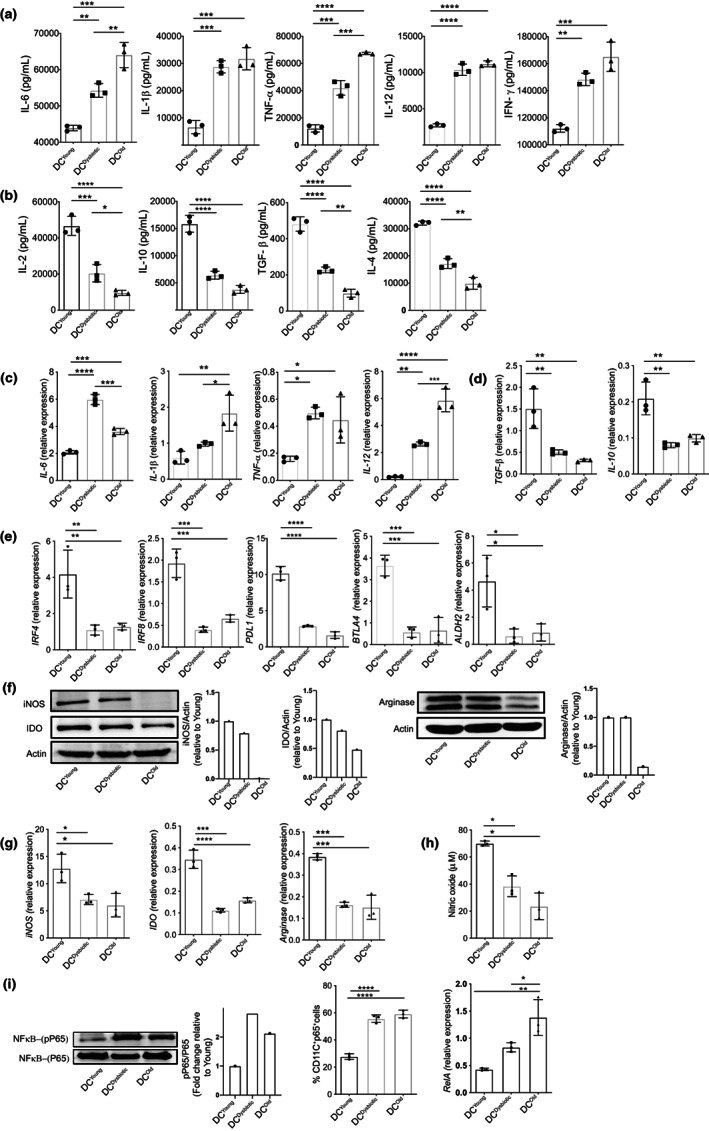
Mechanism of gut dysbiosis‐mediated loss of tolerogenic potential in DCs. Co‐culture supernatants of LPS‐stimulated DC^Young^, DC^Dysbiotic^, and DC^Old^ with syngeneic CD4^+^ T cells were analyzed with ELISA for cytokines secretion; (a) bar graph representation of pro‐inflammatory cytokines IL‐6, IL1‐β, TNF‐α, IL‐12, and IFN‐γ; (b) bar graph representation of anti‐inflammatory cytokines IL‐2, IL‐10, TGF‐β, and IL‐4. qRT‐PCR based quantification of gene expression in DC^Young^, DC^Dysbiotic^, and DC^Old^. Bar graph representation of (c) relative gene expression of pro‐inflammatory cytokine genes; (d) relative gene expression of anti‐inflammatory cytokine genes; (e) relative gene expression of tolerogenic genes *Irf4*, *Irf8*, *Pdl1*, *Btla*, and *Aldh2*. LPS‐stimulated DC SNs were estimated for NO secretion, and the same cells were examined for the expression of tolerogenic metabolic enzymes and phosphorylation status of the p65 subunit of NF‐kB; (f) representative Western blots and protein level quantification of iNOS, IDO, and arginase; (g) relative expression of metabolic genes *iNOS*, *Ido*, and arginase‐1 by qRT‐PCR; (h) bar graph representation of NO secretion by Griess method; (i) representative Western blot and protein level quantification of phospho‐p65 in DCs lysate (left panel), bar graph representation of the percentage of phospho‐p65^+^ DCs through flow cytometry and relative expression of p65 encoding gene *RelA* through qRT‐PCR. Data (mean ± SD) representing RT‐PCR and flow cytometry are of three independent experiments, with each point representing a pool of three animals for one independent experiment, *n* = 3 mice/group, and Western blot data representing two independent experiments. *p < 0.05, **p < 0.01, ***p < 0.001, ****p < 0.0001. Statistical analysis was done by one‐way ANOVA followed by Tukey's multiple comparison test.

We analyzed the supernatant of DC:CD4^+^ T‐cell co‐culture for estimation of pro‐inflammatory and anti‐inflammatory cytokines concentration. A significant increase in pro‐inflammatory cytokines IL‐6, IL1‐β, TNF‐α, IL‐12, and IFN‐γ was noticed when CD4^+^ T cells were co‐cultured with DC^Dysbiotic^ and DC^Old^ compared to DC^Young^, which corroborates with a significant reduction in the anti‐inflammatory cytokines IL‐2, IL‐10, TGF‐β, and IL‐4 release (Figure 2a, b). These results were further validated at the transcript level. We found significantly increased expression of pro‐inflammatory cytokines (*Il‐6*, *Il1b*, *Tnfa*, and *Il‐12*) while reduced transcripts were observed for anti‐inflammatory cytokines *Il‐10*, and *Tgfb* (Figure 2c, d) in DC^Dysbiotic^ and DC^Old^ compared to DC^Young^. Reduced induction of Tregs and decreased production of anti‐inflammatory cytokines strongly supported the curtailed tolerogenic immune responses of DC^Dysbiotic^ and DC^Old^ compared to DC^Young^. These results agree with the studies demonstrating the role of DCs in the induction of peripheral tolerance (Domogalla et al., 2017).

Several studies have demonstrated the role of tolerogenic genes *Irf4*, *Irf8* (McDaniel et al., 2020), *Pdl1* (Oh et al., 2020), *Btla* (Li et al., 2011), and *Aldh2* in regulating the function of DCs (Zhu et al., 2013). To further confirm the tolerogenic dysfunction of young dysbiotic and old DCs, we checked the expression of these genes on matured DCs. A significant decline in the expression of *Irf4*, *Irf8*, *Pdl1*, *Btla4*, and *Aldh2* was noticed in the DC^Dysbiotic^ and DC^Old^ in comparison with DC^Young^ (Figure 2e). In addition, we checked the expression of *Tim3*, an inhibitory receptor (Anderson et al., 2016) on BMDCs through flow cytometry and observed a reduced expression (Figure S2).

Cellular metabolism plays a key role in determining the immunogenic or tolerogenic fate of DCs (Sim et al., 2016). Tryptophan and L‐arginine catabolizing enzymes Indoleamine 2,3‐dioxygenase (IDO) and Arginase I (Arg 1), respectively, cooperate with DCs to confer their immunosuppressive effect (Mondanelli et al., 2017). These catabolizing enzymes deprive T cells of amino acids and lead to their suppression (Grohmann et al., 2003). IDO1 suppresses the allogeneic T‐cell proliferation (Funeshima et al., 2005) and is implicated in the Tregs generation (Fallarino et al., 2006). On the contrary, DC‐derived nitric oxide (NO) determines either the regulatory or effector DC differentiation (Si et al., 2016). NO is synthesized from the metabolism of L‐arginine by inducible NO synthase (iNOS) and induces tolerance in allograft models (Peche et al., 2005). Hence, it was of our interest to determine their role in gut dysbiosis‐mediated DC modulation. We evaluated the protein, and transcript levels of IDO, iNOS, and Arginase1 in DC^Young^, DC^Dysbiotic^, and DC^Old^ stimulated with LPS. A reduction in iNOS, arginase, and IDO at the protein level was observed in DC^Dysbiotic^ and DC^Old^ compared to DC^Young^ through Western blot (Figure 2f); concurrently, their transcript levels were also significantly reduced (Figure 2g). Next, NO was measured in the culture supernatants, and a significant decrease in the production of NO was observed in both DC^Dysbiotic^ and DC^Old^ compared to DC^Young^ (Figure 2h) complementing the decreased NO metabolizing enzyme, iNOS.

### 2.4 Gut dysbiosis due to aging and antibiotic treatment of young mice induces the NF‐κB pathway in DCs to regulate their immunogenic/inflammatory phenotype

To decipher the molecular mechanism that plays a role in influencing the tolerogenic properties of DCs upon gut dysbiosis, we investigated NF‐κB signaling pathway. This pathway has a quintessential role in regulating DC tolerance as it is the master regulator of various genes involved in the DC maturation (Ade et al., 2007). Inhibiting NF‐κB signaling has been reported to differentiate and maintain tolerogenic DCs in the context of cancer, autoimmune disorders, and aging (Carreno et al., 2011). First, to quantify NF‐κB activation, we checked the phosphorylation status of p65, a DNA binding subunit through Western blot and flow cytometry. An increase in phosphorylation of p65 was observed in DC^Dysbiotic^ and DC^Old^ compared to DC^Young^ (Figure 2i, left and middle panel). Furthermore, the mRNA expression of *RelA* encoding for RelA/P65 in total DC extract was observed significantly augmented in young dysbiotic and aged DCs compared to DC^Young^ (Figure 2i, right panel). These results suggested an activated NF‐κB phenotype of DCs and the possible role of gut microbiota in the functional modulation of DCs.

### 2.5 Gut dysbiosis influenced by aging alters the abundance of the genus *Lactobacillus* in the gut, and its replenishment with *Lactobacillus plantarum* restores dysbiosis and DC phenotype

**FIGURE 3:**
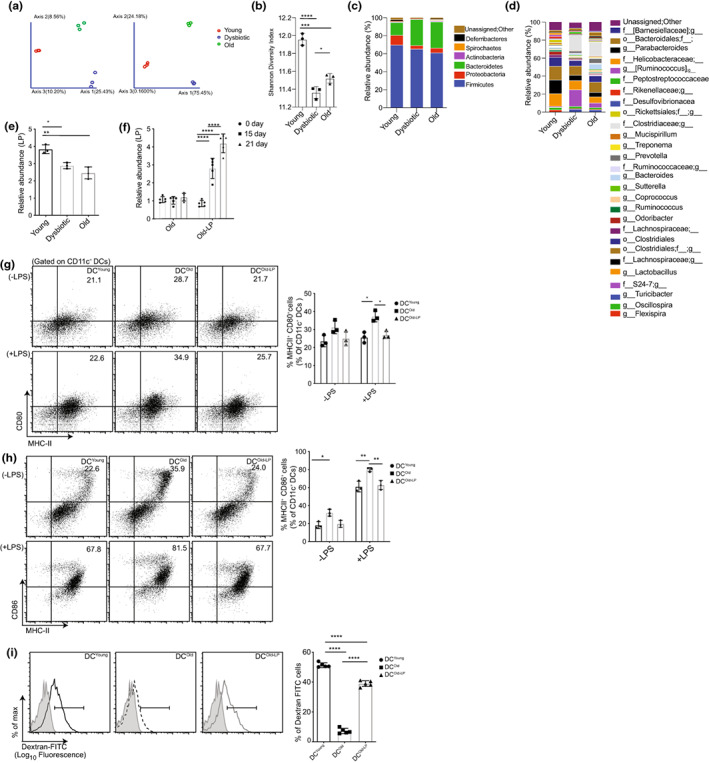
Gut microbiota alteration with aging and antibiotics exposure and evaluation of the immunomodulatory role of *Lactobacillus plantarum*. The fecal DNA of young control, young dysbiotic (21 days of antibiotics treated), and old mice were analyzed with 16S rRNA sequencing and qRT‐PCR; (a) adapted weighted and unweighted unifrac PCoA plots indicating differential beta diversity; (b) Shannon diversity index depicting alpha diversity; (c) bar graphs represent relative abundance at the phylum level; (d) bar graphs represent relative abundance at the genus level; (e) bar graph represents qRT‐PCR data for the indigenous relative abundance of species *L. plantarum*; (f) bar graph represents qRT‐PCR based quantification of relative accumulation of *L. plantarum*on the day 0, 15 and 21 of administration through oral gavage with (10^8^ CFU/mL) in old mice; (g) dot plot and bar graph depicting percentage population of MHCII^+^CD80^+^ DCs; (h) MHCII^+^CD86^+^ DCs gated on CD11c^+^ cells and (i) Representative histogram and frequency of dextran‐FITC uptake, as assessed by flow cytometry in DC^Young^, DC^Old^ and DC^old‐LP^. All the data (mean ± SD) are from three independent experiments, with each point representing a pool of three animals for one independent experiment, *n* = 3 mice/group except (f, i), with each point in the bar graph indicating one animal, *n* = 5. The statistical analysis done for RT‐PCR (f) and flow cytometry data (g, h) is by two‐way ANOVA followed by Sidak's multiple comparisons test, and the rest is by one‐way ANOVA followed by Tukey's multiple comparison test. *p < 0.05, **p < 0.01, ***p < 0.001, ****p < 0.0001.

Next, we were curious to decode the involvement of the gut microbiota in age‐predisposed dysbiosis and loss of DC tolerance. We observed a significant difference in the overall microbiota composition of young dysbiotic and old mice compared to young mice. The beta diversity plot which measures the phylogenetic relationship of bacterial communities between groups through both weighted and unweighted principal coordinate analysis indicated separate clustering among all three groups, suggesting a difference in microbial communities (Figure 3a). Alpha diversity analysis which measures the species richness and evenness within an ecological community through Chao1 (community richness) and Shannon index (evenness) (Hughes et al., 2001) showed distinct measures among the group. Both indices indicated a gradual decrease in diversity and evenness of microbiota in young dysbiotic and old mice groups. The Chao1 diversity index revealed a decreased species richness in young dysbiotic and old groups of mice compared to the young group, but the difference was not significant (Figure S3a). However, a significant decrease was noted in the Shannon diversity index (Figure 3b), indicating a biased microbial community structure in old and young dysbiotic mice. Further, phylum and genus level comparisons for relative abundance among all three groups were done. Phylum, like Firmicutes showed a substantial decrease in young dysbiotic and old mice with a concurrent increase in phylum Bacteroidetes with respect to the young group (Figure 3c). At the genus level within phylum Firmicutes, beneficial genera like *Lactobacillus* and the genus of the family Lachnospiraceae expressed a significant drop while harmful bacterial genera like *Turicibacter* and the genus of the family Clostridiaceae showed a substantial increase in abundance with the age and Abx treatment of young mice (Figure 3d). The differential abundance in young dysbiotic and old mice in comparison with young was also confirmed by linear discriminant analysis, as depicted in LEfSe plots (Figure S3b).

In our analysis, the diversity plot showed significant downregulation of the genus *Lactobacillus* in both old and young dysbiotic groups. *Lactobacillus* strains maintain immune tolerance by inhibiting inflammatory immune responses (Esmaeili et al., 2018; Macho Fernandez et al., 2011) of macrophages and epithelial cells (Ferreira Dos Santos et al., 2016) and by induction of Tregs (Bermudez‐Brito et al., 2018). Consequently, we were intrigued to explore the role of *Lactobacillus* in restoring the function of DCs. First, we measured the abundance of *Lactobacillus plantarum* (LP) in fecal samples of all three groups of mice and observed a significant reduction in LP abundance with age and antibiotic treatment of young mice compared to young mice (Figure 3e). Next, we obtained *Lactobacillus plantarum* (LP) (MTCC‐2621 corresponding to ATCC‐8014) from MTCC‐CSIR‐IMTECH and replenished the old mice group. We observed a gradual accumulation of LP in the gut of inoculated mice (Old‐LP) from Days 15 to 21 (Figure 3f). Age‐associated microbial dysbiosis promotes increased intestinal permeability leading to systemic inflammation and innate immune system dysfunction (Thevaranjan et al., 2017). Therefore, we first measured the effect of LP on the restoration of intestinal permeability. LP‐inoculated old mice showed a significant decrease in gut permeability compared to young dysbiotic and old mice as evidenced by a lower level of dextran FITC in blood plasma (Figure S3c). As LP was able to recover the intestinal permeability, we hypothesized that LP inoculation and establishment in the gut can circumvent the systemic inflammatory condition, by modulating bone marrow myeloid progenitors to acquire tolerogenic properties. Hence, we investigated the effect of LP administration on the phenotype and functional properties of the DCs of LP‐inoculated old mice (DC^Old‐LP^). We observed a significant decline in the display of the MHCII and co‐stimulatory molecules CD80, CD86, and CD40 (Figure 3g, h and Figure S3d) and an increase in co‐inhibitory molecule Tim‐3 (Figure S3d, lower panel) on DC^Old‐LP^ compared to DC^Old^. LP plays a critical role in regulating immune response by modulating the expression of anti‐inflammatory cytokines such as IL‐10 and TGF‐β (Lamubol et al., 2021). Once we had the evidence of phenotypic restoration, we intended to investigate the LP modulation on secretory components of old DCs. We detected a significant reduction in pro‐inflammatory cytokines (IL‐6, TNF‐α, IL‐12, IFN‐γ, IL1‐β, and IL‐17) and an increase in anti‐inflammatory cytokines (IL‐2, IL‐10, TGF‐β, IL‐4) in the CD4^+^ T cell: DC^Old‐LP^ co‐culture in comparison with DC^Old^ (Figure S3e). These results were further validated by transcript‐level analysis. Concurrently, significantly decreased transcript levels of pro‐inflammatory cytokines (*Il‐6*, *Il‐12*, *Il‐23*, *Il1b, Tnfa*) and increased transcripts of anti‐inflammatory cytokines (*Il‐10* and *Tgfb*) (Figure S3f) genes were observed in DC^Old‐LP^. Intriguingly, our observation further got strengthened with the results showing upregulation in the transcript levels of *Irf4*, *Irf8*, *Pdl1*, *Pdl2*, *Btla4*, and *Aldh2* tolerogenic genes in DC^Old‐LP^ (Figure S3g). Next, we checked the effect of LP on functional properties of DCs and assessed the phagocytic capacity of replenished DC^Old‐LP^, an increase in phagocytosis of dextran‐FITC and apoptotic Jurkat cells in old‐LP BMDCs was observed (Figure 3i and Figure S3h).

### 2.6 *Lactobacillus plantarum* modulates the tolerogenic regulatory program of old DCs by metabolic rewiring and downregulation of NF‐κB pathway

**FIGURE 4:**
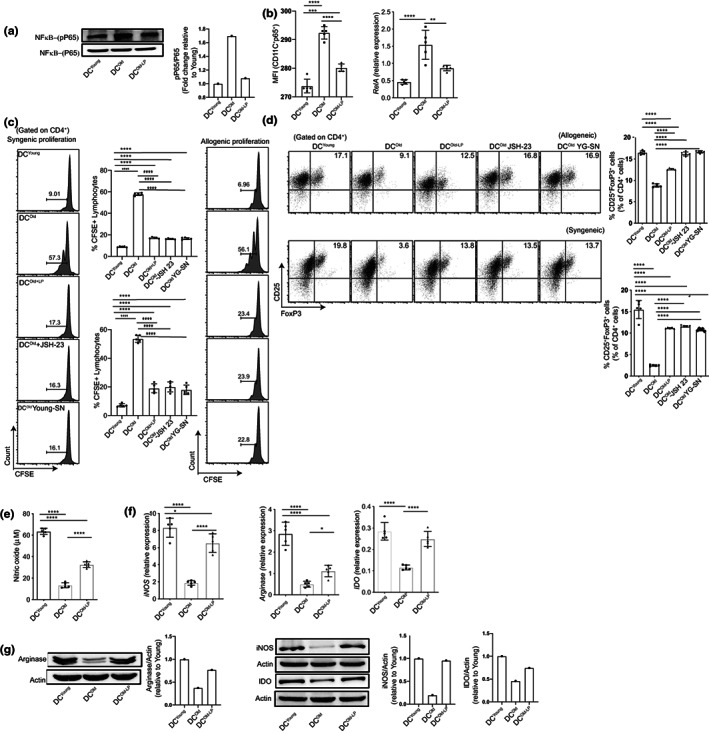
Replenishment of the gut of old mice with *Lactobacillus plantarum* reinstates the tolerogenic and metabolic function of old DCs with downregulation of the NF‐kB pathway. Evaluation of LPS‐stimulated DC^Young^, DC^Old^, and DC^Old‐LP^ for NF‐kB pathway activation, metabolic gene modulation and their functional properties; (a) representative Western blot and protein level quantification of phospho‐p65 in DCs lysate; (b) bar graph representation of the frequency of phospho‐p65^+^ DCs through flow cytometry and relative expression of p65 encoding gene *RelA* through qRT‐PCR; (c) representative histogram and frequency of proliferative CD4^+^ T cells in syngeneic and allogeneic setup; (d) representative dot plots and frequency of Tregs (CD25^+^FoxP3^+^) gated on CD4^+^ T cells in different groups with allogeneic and syngeneic setup; (e) quantification for the concentration of NO released by LPS‐stimulated DCs in SN as determined by the Griess method; (f) relative gene expression of *iNOS*, *arginase1*, and *Ido1* evaluated by qRT‐PCR; (g) representative Western blot and quantification of iNOS, IDO, and arginase1 at the protein level. Data (mean ± SD) are of 5 animals, with each point in the bar graph representing one animal, *n* = 5 except (a, g) data representing one independent experiment. *p < 0.05, **p < 0.01, ***p < 0.001, ****p < 0.0001. One‐way ANOVA followed by Tukey's multiple comparison test was performed for statistical analysis.

*Lactobacillus plantarum* has been shown to modulate NF‐κB mediated inflammatory pathway in the model of colitis (Yu et al., 2020) and bacterial pathogenesis (K. Li et al., 2022). Hence, we revisited NF‐κB signaling and metabolic pathways involved in the loss of tolerogenic properties of old‐DCs to evaluate the potential of LP for restoration of tolerance. Replenishing the gut of old mice with LP significantly reduced the phosphorylation of p65 in DC^Old‐LP^ as evident from the Western blot and flow cytometry compared to DC^Old^ (Figure 4a, b), with a concomitant decline in *RelA* expression in LPS‐stimulated DC^Old‐LP^ compared to DC^Old^ while their level was comparable with DC^Young^ (Figure 4b, right panel). Next, we intended to examine the impact of LP replenishment in regulating DC‐mediated T‐cell proliferation through attenuation of NF‐κB signaling. We used NF‐κB inhibitor JSH‐23 (Wu & Li, 2012) to block its translocation in the nucleus, and parallelly supernatant of DC^Young^ with attenuated NF‐κB signaling was used (shown in Figure 2i) in further experiments. We observed significant inhibition of both syngeneic and allogeneic CD4^+^ T‐cell proliferation in DC^old‐LP^, DC^Old^ with JSH‐23, and DC^Old^ co‐culture suspended in DC^Young^ cell‐free supernatant compared to DC^Old^ (Figure 4c). Additionally, a significant alleviation in Tregs conversion with NF‐κB attenuation was noticed (Figure 4d). These results confirmed our led hypothesis that the age‐mediated tolerogenic defect of old DCs emanates from secretory factors in the microenvironment. Our experimental evidence shows that the pro‐inflammatory microenvironment is the driving factor of deregulated NF‐κB pathway in old DCs. Upon correction of the microenvironment with young DCs supernatant, tolerogenic properties can be restored.

We further substantiated our results by examining metabolic moieties responsible for tolerogenic behavior of DCs. DC^old‐LP^ exhibited a significant increase in the secretion of NO (Figure 4e) with elevated expression of *iNOS*, *arginase1*, and *Ido* genes as compared to DC^old^ (Figure 4f). We further confirm their increase at protein levels by Western blotting (Figure 4g). Dendritic cells that exhibit tolerogenic behavior normally engage in the mitochondrial OXPHOS for energy. After activation, they become immunogenic and switch towards glycolysis, akin to Warburg metabolism (Krawczyk et al., 2010). Accordingly, we found it a factor of relevance whether LP replenishment is also modulating the same. Additionally, glucose uptake and associated transporter molecules could be the related factors, that directly regulate this process (Cho et al., 2017). Hence, we monitored 2‐NBDG (2‐(N‐(7‐Nitrobenz‐2‐oxa‐1,3‐diazol‐4‐yl) Amino)‐2‐Deoxyglucose) uptake in both BMCs and BMDCs by flow cytometry. A significant reduction in 2‐NBDG uptake was observed in both DC^Young^ and DC^Old‐LP^ in comparison with DC^Old^ and DC^Dysbiotic^ (Figure S4). Further, we evaluated the expression of the *Slc2a1* gene, encoding GLUT1, a known glucose transporter, and observed lesser expression in DC^Old‐LP^ (Figure S4c). These results suggest that LP fixes the metabolic network to promote tolerance in DC^Old‐LP^. These results suggest that administration of LP in the gut not only restores the phenotype and the features of DC^Old^ but also reinvigorates their functional properties to the level of DC^Young^.

### 2.7 *Lactobacillus plantarum* modulates aged DCs by enhancing their migratory and regulatory function

**FIGURE 5:**
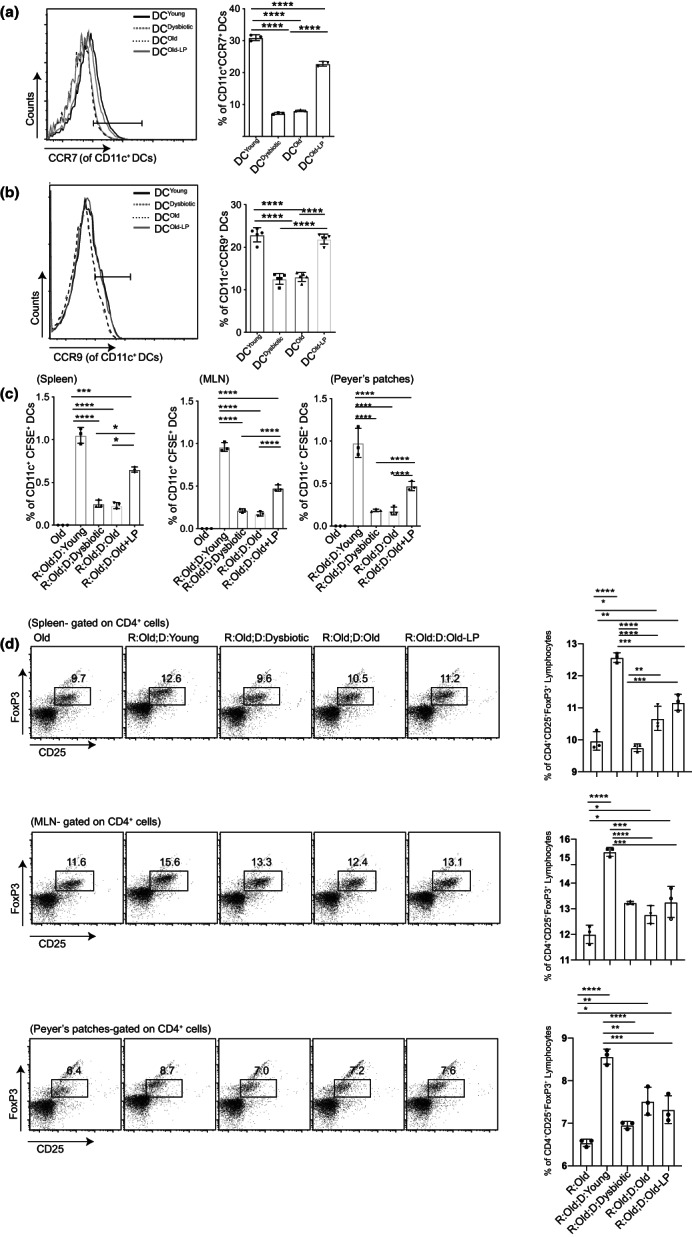
*Lactobacillus plantarum* reinvigorates the migratory and regulatory function of Old‐ DCs. LPS‐stimulated DC^Young^, DC^Dysbiotic^, DC^Old^, and DC^Old‐LP^ were estimated for in‐vitro estimation of migratory markers. Representative histogram and frequency of (a) CCR7^+^ CD11c^+^ cells; (b) CCR9^+^ CD11c^+^ cells through flow cytometry. Estimation of in vivo migration and Tregs inducing capacity of adoptively transferred LPS‐stimulated DC^Young^, DC^Dysbiotic^, DC^Old^, and DC^Old‐LP^; (c) bar graph representation of the frequency of CFSE^+^ CD11c^+^ (R: Recipient, D: donor), and (d) representative dot plot and bar graph representation of CD25^+^Fox P3^+^Tregs (gated on CD4 cells) in the spleen, MLN and Peyer's patches. Data (mean ± SD) are of 3 animals, with each point in the bar graph representing one mouse (*n* = 3/group). *p < 0.05, **p < 0.01, ***p < 0.001, ****p < 0.0001. Statistical analysis was done by one‐way ANOVA and Tukey's multiple comparison test.

The regulatory role of DCs depends on their ability to migrate to the draining lymph nodes to activate T cells (Hadeiba et al., 2008). CCR7 and CCR9 are important migratory molecules present on the DC surface, driving their trafficking in the skin and gut, respectively, to induce tolerance (Ohl et al., 2004; Pathak et al., 2020). Therefore, we studied the expression of these molecules and DC migration in vivo. A significant decline in the expression of both CCR7 and CCR9 was observed on DC^Dysbiotic^ and DC^Old^ compared to DC^Young^; however, their expression was restored significantly upon LP administration on DC^Old‐LP^ (Figure 5a, b). Next, we checked the migratory capacities of adoptively transferred DCs from all four groups in old mice in the spleen, mesenteric lymph nodes (mLN), and Peyer's patches (PP). In the same group of mice, the regulatory functions of these migrated DCs were evaluated by analyzing the Tregs induction. We observed a significant decrease in the migratory ability of DC^Old^ and DC^Dysbiotic^ compared to DC^Young^, while restoration of migration was observed upon LP administration in all three examined organs: spleen, mLN, and PP (Figure 5c). Likewise, shrinkage in the pool of Tregs was detected which was reinvigorated in the LP‐inoculated old animals (Figure 5d). Our results showed that DC^Young^ is a potent inducer of Tregs in all three organs (spleen, mLN, and PP) under inflammatory conditions (old recipient), whereas DC^Dysbiotic^ and DC^Old^ significantly lose their Tregs induction potential compared to young DCs. These experiments suggest that due to the enhanced migratory ability, DC^Young^ and DC^Old‐LP^ induced Tregs differentiation and thereby induced tolerance.

### 2.8 *Lactobacillus plantarum* modulates the age‐associated gene expression profile of DC^Old^ and directs them towards a tolerogenic phenotype

**FIGURE 6:**
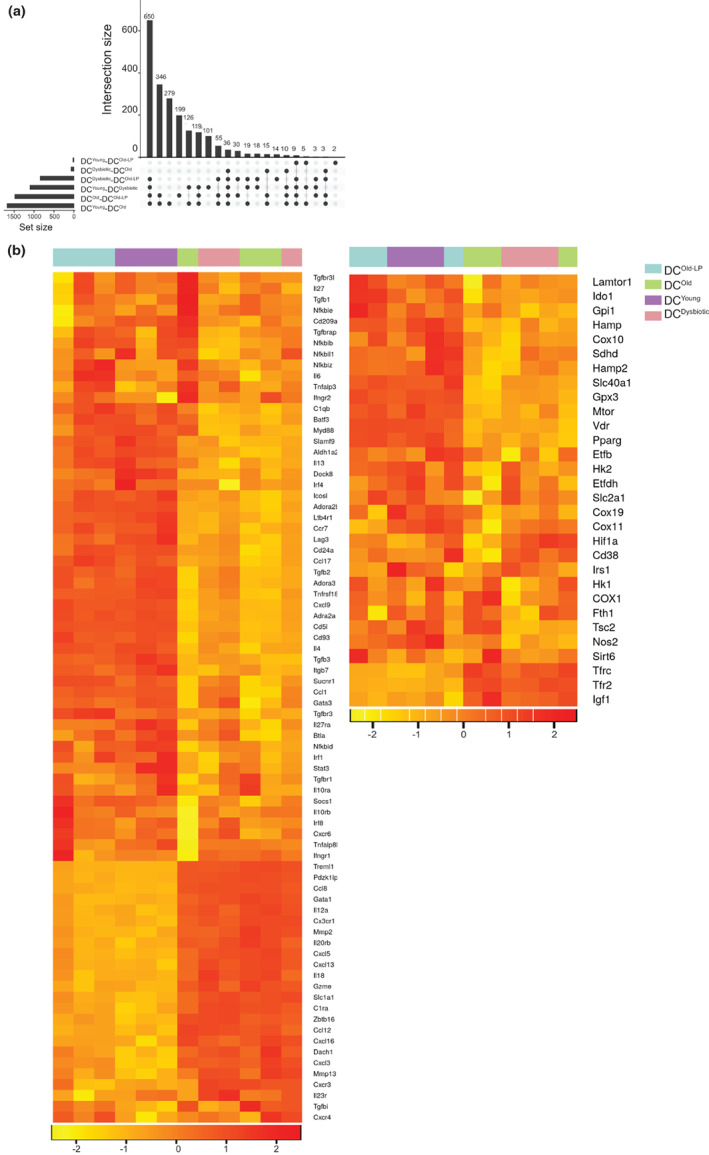
Inoculation of *Lactobacillus plantarum* into the gut of old mice modulates the transcriptomic gene profile. RNA was isolated from LPS‐stimulated DC^Old‐LP^, DC^Old^, DC^Dysbiotic^, and DC^Young^ for gene array profiling by Agilent GeneChips. Data analysis was done, and differential gene expression between the groups was compared. (a) UpSet plot representing the intersection between the sets of differentially expressed genes from various comparisons in the limma setup with a vertical bar plot reporting the intersection size, the dot plot was reporting the set participation in the intersection, and the horizontal bar plot reporting the set sizes. The heatmap (b) represents the differentially expressed features associated with the immune response (left panel), differentially expressed metabolism‐related features (right panel). Rows represent the features, and columns signify the samples. The ‘Color Key’ denotes the log‐transformed expression values. Data are from three independent sets, with each data set representing a pool of three animals (*n* = 3 mice/group).

*Lactobacillus* influence the physiology of their hosts by diverse mechanisms, specifically by interacting with the immune system and play an imperative role in the development and maintenance of the same (Kemgang et al., 2014; van Baarlen et al., 2009). To unravel the molecular mechanism of LP‐mediated immunomodulation, we performed the global transcriptomic profiling of DC^Young^, DC^Old^, DC^Dysbiotic^, and DC^Old‐LP^ groups. The differential gene expression analysis was filtered by logFC cutoff of 0.5 and a significant *p* value cutoff threshold of <0.05. Our data revealed a contrasting similarity between DC^Dysbiotic^ with DC^Old^ and DC^Young^ with DC^Old‐LP^ as depicted in volcano plots (Figure S5a). Maximum significantly differentially expressed (DE) features were observed between DC^Young^ and DC^Old^ pair (1673), and importantly least DE features were obtained among DC^Young^ and DC^Old‐LP^pair (19), DC^Old‐LP^ exhibit intermediate significant DE features when compared with DC^Old^ (1478) and DC^Dysbiotic^(839), which suggests that LP replenishment rewires the transcriptome profile of old‐LP DCs. The intersection of differentially expressed gene sets is represented as an UpSet plot (Marwah et al., 2019) which infers overall larger variation among DC^Young^ with DC^Old^ and DC^Dysbiotic^ groups while there was little variation with DC^Old‐LP^. Similar observations were made for the comparison of DC^Old^ with DC^Dysbiotic^ (Figure 6a). On further analysis differentially expressed genes were grouped under three broad categories of immune response, metabolism, and cell cycle‐DNA repair pathways (Figure 6b and Figure S5b). Tolerogenic genes such as *Tgfb*, *Lag3*, *Irf4*, *Irf8*, and *Aldh1a2* were found upregulated in DC^Old‐LP^, and similar patterns were observed for metabolic genes with significant fold change in DC^Old^ versus DC^Old‐LP^ with upregulation of genes encoding immune tolerance enzymes as Ido1, *CD38*, and *NOS2* with least fold difference in DC^Young^ versus DC^Old‐LP^ (Figure 6b). Furthermore, in the case of DNA damage‐associated genes, most of them showed increased fold change expression in DC^Young^ and DC^Old‐LP^ compared to DC^Old^ such as *Cdc20*, *Cdk2*, *Pik4*, *Pik2*, and *Parp* group of genes 1, 3, 4, and 11 having an important role in the recovery of cells from DNA damage (Figure S5). All these findings indicate the substantial role of aging and antibiotic‐mediated gut dysbiosis towards the generation of inflammatory phenotype, which interestingly resorted towards tolerogenic mode by replenishment of gut of old mice with LP.

## 3 DISCUSSION

Aging is an incessant and irrevocable physiological process accompanied by the phenotypic and functional changes of senescence. It results in chronic inflammation, the decline in functional immunity, and altered gut microbial composition. The gut microbiome profoundly influences human health and disease, and recently, gut dysbiosis has been connected with several chronic diseases viz. inflammation, neurodegenerative diseases, metabolic disorders, and cancer (Belkaid & Hand, 2014; Zheng, Fang, et al., 2020). However, the mechanism of age‐associated immune dysfunction through gut dysbiosis remains largely unexplored. Consequently, in the current study, we have tried to uncover the influence of aging on gut microbiota and its impact on DCs. Additionally, we explored the prospect of a key probiotic strain for restoring age‐mediated DC dysfunction.

Dendritic cells play an essential role in regulating immunity and tolerance by connecting innate and adaptive immune systems (Raker et al., 2015). Tolerogenic DCs maintain central and peripheral tolerance by regulation of optimum effector T cell and regulatory T‐cell responses (Hasegawa & Matsumoto, 2018). However, with age, the regulatory function of DCs is impaired. The current study explores the gut‐age axis by monitoring dysbiosis‐associated functional defects of dendritic cells (DCs). We did a comparative analysis of the gut microbiome of young animals with aged and gut‐disrupted young mice and simultaneously compared the DCs from all of them for their phenotypic characteristics and functional capacity. As compared to the young group (DC^Young^), the DCs generated from aged (DC^Old^) and young gut‐disrupted animals (DC^Dysbiotic^) exhibited the following differences: (i) loss of tolerance, as supported by upregulation in the level of pro‐inflammatory molecules IL‐6, IL‐1β, TNF‐α, IL‐12, IFN‐γ, NF‐κB, p65, RELA, and decline in the anti‐inflammatory moieties IL‐2, IL‐10, TGF‐β, IL‐4, IDO, arginase, NO, IRF‐4, IRF‐8 PDL1, BTLA4, and ALDH2; (ii) impaired functionality, as evinced by the failure to control the hyperactivation of T cells; (iii) inability to generate the optimum frequency of Tregs; (iv) contraction of *Lactobacillus* from the gut of the old and Abx treated young mice; and (v) replenishment of gut of old mice with *Lactobacillus plantarum* restored the tolerogenic phenotype and function of old DCs.

We observed that NF‐κB signaling was required for the homeostatic maturation and function of DC^Young^ while overactivation was observed in DC^Dysbiotic^ and DC^Old^. NF‐κB is a master regulator of inflammation, and its dysregulated activation is associated with several autoimmune, inflammatory pathogenesis (Barnabei et al., 2021) and aging (Adler et al., 2007). Over‐expression of NF‐κB subunit RelA/p65 induces a senescent phenotype in cultured cells, while the low level in the progeroid mouse model has been interrelated with the delay in age‐associated pathologies (Flores et al., 2017; Seitz et al., 2000). In agreement, we demonstrate that overt activation of NF‐κB in DC^Dysbiotic^ and DC^Old^ is due to increased phosphorylation of p65, interestingly, p65 phosphorylation decline in DC^Old‐LP^ on feeding the old animals with LP. We assume that the decline in phosphorylation of p65 in DC^Old‐LP^ is associated with secreted factors, as an inhibitor of p65 (JSH‐23) and supernatant of DC^Young^ culture show a similar effect on DC^Old^ for NF‐κB downregulation through p65 phosphorylation. Increased activation of NF‐κB was associated with the maturation status of old and dysbiotic DC. In comparison with DC^Young^ both DC^Dysbiotic^ and DC^Old^ exhibited heightened expression of co‐stimulatory and MHC molecules and decrease in the level of inhibitory molecules. Further, reduced phagocytosis was noticed, since DC^Dysbiotic^ and DC^Old^ poorly captured antigens and apoptotic bodies. Our findings further suggest that gut dysbiosis affects the tolerogenic properties of DC as enhanced CD4^+^ T‐cell proliferation, increased release of pro‐inflammatory cytokines and reduction in the pool of Tregs and the level of anti‐inflammatory cytokines were observed. Unregulated T‐cell proliferation upon aging has been linked previously to loss of DC tolerance (Agrawal & Gupta, 2011). Since, the secretion of anti‐inflammatory cytokines and reduced secretion of pro‐inflammatory cytokines by DC contribute to tolerance induction (Wakkach et al., 2003). We conclude that gut dysbiosis leads to the activation of NF‐κB signaling in DC and activates pro‐inflammatory phenotype conducive for CD4^+^ T‐cell activation. Interestingly, LP reconstitution restored the phenotype and functional properties of old DCs as evident by optimum expression of co‐stimulatory and MHCII expression on DC^Old‐LP^, improved phagocytic activity and controlled proliferation of CD4^+^ T cells.

Our analysis of the gene expression signature through global transcriptomic profiling of all four groups of DCs reveals a striking difference between DC^Young^ and DC^Old^ where the later is similar to DC^Dysbiotic^ for gene expression. Interestingly, LP reconstituted old DC (DC^Old‐LP^) aligned with DC^Young^ in the molecular screening. A convincing repression of NF‐κB inhibitor genes was observed in both DC^Dysbiotic^ and DC^Old^ compared to DC^Young^. Activation of NF‐κB genes coincided with the repression of *Tgfb* genes and receptors. TGFβ has been linked with tolerogenic function in DC (Zhong et al., 2019), and transcriptional activation of *Smad7* in response to pro‐inflammatory cytokine stimulation through NF‐κB activation has been shown to suppress TGFβ/SMAD signaling (Bitzer et al., 2000). In our experimental settings, we have shown downregulation of *Tgfb* expression, and the corresponding decrease in TGFβ in the supernatant of DC^Old^ and DC^Dysbiotic^ CD4^+^ T‐cell co‐culture. Our transcriptomic profiling re‐confirmed our results; we observed downregulation of *Tgfb*, *Tgfb* receptors and associated receptors (Tgfb1, Tgfr3, and Tgfbrap1) in DC^Old^ and DC^Dysbiotic^ compared to DC^Young^. LP reconstitution restores the TGFβ signaling by downregulating the NF‐κB activation.

The tolerogenic phenotype of DCs to induce T‐cell hypo‐responsiveness is orchestrated by a complex network of immunoregulatory molecules (Domogalla et al., 2017) and metabolic rewiring (Adamik et al., 2022). Transcription factors IRF4, IRF8, and BATF3 have been shown to promote tolerogenic phenotype of DCs by promoting Tregs generation through negative regulation of pro‐inflammatory cytokine transcription and induction of *Raldh*, *Pdl1*, and *Pdl2* expression (Vander Lugt et al., 2017), and prevention of inflammasome activation in splenic DCs (McDaniel et al., 2020). Likewise, we observed a downregulation in the expression of *Irf4*, *Irf8*, *Pdl1*, *Btla*, and *Aldh2* with a potent reduction in Treg inducing capacities of both DC^Dysbiotic^ and DC^Old^ but not DC^Young^. Intriguingly, LP replenishment restores the tolerogenic properties of DCs by upregulating the expression of immunoregulatory molecules. The Treg induction was boosted, and it coincided with enhanced migratory capacity of DCs. Further, the downregulation in the pattern of NO, iNOS, arginase 1 and IDO in DC^Dysbiotic^ and DC^Old^ that contributed to their immunogenic phenotype was also restored through LP reconstitution. The NO, iNOS, arginase 1, and IDO have been implicated in imparting tolerogenic behavior of DC (Panfili et al., 2019; Verinaud et al., 2015). NO responsible for the tolerogenic behavior of DCs is a product of L‐arginine metabolism and its level is controlled in cells by iNOS (Moncada, 1999) while immunoregulatory enzyme IDO elicits peripheral tolerance in DCs. Another important observation in this study included the rewiring of metabolic processes in DC^Old^ by switching from mitochondrial OXPHOS to a glycolytic pathway with greater consumption of glucose compared to DC^Young^ and DC^Old‐LP^. Notably, DCs at a steady‐state condition showing tolerogenic behavior normally engage in mitochondrial OXPHOS for energy requirements. However, after TLR‐based activation they become immunogenic with the remarkable switch towards glycolysis, similar to Warburg metabolism (Krawczyk et al., 2010).

Overall, the parallel observations in DC^Dysbiotic^ and DC^Old^ suggest that disruption in gut microbiota due to aging or antibiotic treatment of young mice can contribute to the loss of tolerance in DCs. We have uncovered a notable contribution of gut microbiota contributing to the loss of immune tolerance in the dendritic cells on aging. Our data show a significant role of NF‐κB signaling in immune tolerance at old age, through the regulation of cytokine signaling. Furthermore, disruption of the gut on aging or antibiotic treatment of young group also results in the disappearance of beneficial commensals like *Lactobacillus plantarum*, resulting in the loss of tolerance in DCs. Interestingly, *LP* replenishment restores tolerogenic phenotype and properties of old DCs through modulation of metabolism and immunity‐associated gene network profiling. The study for the first time demonstrates the role of aging on gut dysbiosis, which ultimately resulted in the loss of functionality in DCs in maintaining tolerance. Finally, this study suggests the immunotherapeutic role of LP to treat age‐related disruption in the gut for the tolerogenic function of dendritic cells.

## 4 METHODS

### 4.1 Animals and ethical statement

Animal experiments were performed with C57BL/6 female mice. 2–3 months old mice were considered young and 22–24 months were considered old. All the animals were procured from IMTECH Center for Animal Resources and Experimentation (iCARE), Institute of Microbial Technology (IMTECH), Chandigarh. Institutional Animal Ethics Committee (No. 55/1999/CPCSEA), Ministry of Environment and Forest, Government of India has approved the study and all the experiments and protocols are in accordance with the guidelines of the same.

### 4.2 Gut dysbiosis mice model

Young mice were given drinking water with a broad‐spectrum antibiotic (Abx) cocktail (Ampicillin, 1 g/L; neomycin sulfate, 1 g/L; metronidazole, 1 g/L and vancomycin, 0.5 g/L) Himedia (Mumbai, India) ad libitum in drinking water for 21 days to disrupt the gut microbiota, with change in Abx containing water after every 3 days. To assess the effect of Abx‐mediated dysbiosis on the gut bacterial burden, serially diluted fecal samples from all groups were plated on a BHI medium under aerobic and anaerobic conditions. Facultative anaerobic conditions were maintained by keeping the plates in airtight sealed glass chambers in presence of Anaerogas packs‐LE002A‐5NO, Himedia (Mumbai, India).

### 4.3 Chemicals and reagents

All reagents were acquired from Sigma Aldrich (St. Louis, MO) or else specified. All recombinant cytokines were purchased from BD Biosciences (San Diego, CA). Fluorochrome‐tagged Abs viz. CD11c‐PEcy7 (#558079), MHCII‐PerCP‐efluor710 (#46–5320‐82), CD86‐PE (#12–0862‐85), CD80‐FITC (#553768), CD40‐ PerCPcy5.5 (#124623), Tim3‐APC (#134007), CCR7‐APC (#120107), and CCR9‐PE (#565576) were procured from BD Biosciences (San Jose, CA), eBioscience (San Diego, CA), and Biolegend (San Diego, CA). Secondary anti‐mouse and anti‐rabbit Abs for flow cytometry and confocal microscopy were from Invitrogen‐Thermo Fisher Scientific (Waltham, MA). ELISA reagents and Abs against IL‐6, IFN‐γ, IL‐17, IL‐12, TGF‐β, IL‐4, TNF‐α, IL‐2, and IL‐10 were purchased from BD Biosciences (San Diego, CA). Primers for qRT PCR, were synthesized by Sigma Aldrich (St. Louis, MO) and Integrated DNA Technologies, Inc. (Coralville, IA). Anti‐mouse phospho p65 (# 3033), p65 (# 8242), iNOS (# 13120), and β‐actin (# 3700) Abs were from Cell Signalling Technology (Danvers, MA), anti‐mouse IDO (# sc‐137,012) from Santa Cruz Biotechnology, (Santa Cruz, CA) and anti‐mouse arginase1 (# 610709) from BD Biosciences, (San Diego, CA) were used in Western blot. Other chemicals and reagents like RPMI‐1640, fetal bovine serum were from GIBCO (Grand Island, NY), l‐glutamine, l‐pyruvate, streptomycin and penicillin‐ from Serva (Heidelberg, Germany). All tissue culture‐grade plasticwares were from BD Biosciences (San Diego, CA), Corning™ (Corning, NY) and Nunc™ (Rochester, New York).

### 4.4 Bacterial strains used in the in vivo experiments

*Lactobacillus plantarum* MTCC 2621 obtained from Microbial Type Culture Collection (MTCC), IMTECH (Chandigarh, India) was cultured in DeMan, Rogosa Sharpe broth (Merck, Darmstadt, Germany) at 37°C, 5% CO_2_. The bacterial suspension was pellet down by centrifugation at 3000 *g* for 10 min and washed with 1xPBS twice, and CFU was adjusted to 1 × 10^8^. Thereafter, the same CFU in 200 μL sterile PBS was orally gavaged to mice every alternate day for 21 days, until mice were sacrificed. Fecal samples were obtained on Days 0, 7, and 21 to assess the accumulation of bacteria inside the gut.

### 4.5 Dendritic cell cultures

Bone marrow cells from the femurs and tibia of mice were flushed with cold RPMI media. After RBC lysis by ACK lysis buffer, the cells (2 × 10^6^/well) were plated in a 6‐well plate. The BMCs were cultured in RPMI‐1640 supplemented with 10% FCS, penicillin (100 U/mL), l‐glutamine (100 mM), streptomycin (100 mg/mL), rmGM‐CSF (2 ng/mL) (#554586) and 4 ng/mL rmIL‐4 (#‐550067), BD Biosciences (San Diego, CA), for DC cultures. On 3rd day, the culture was supplemented with fresh complete RPMI media with rmGM‐CSF (2 ng/mL) and rmIL‐4 (4 ng/mL) at half of its initial volume. Loosely adherent cells were harvested by gentle pipetting on the 7th day and used for further experiments. The methodology was followed as per the already mentioned protocol (Lutz et al., 1999). Later, for maturation of BMDCs (2 × 10^6^/well) were stimulated with LPS‐1 μg/mL, L2630‐ Sigma Aldrich (St. Louis, MO) in 6‐well plate.

### 4.6 Flow cytometry

Single‐cell suspension was incubated for 30 min at 4°C with Fc block (anti‐mouse CD16/32 Ab) to prevent non‐specific binding of Abs. Next, cell‐surface staining was done with fluorochrome‐tagged monoclonal antibodies for another 30 min at 4°C as per the experiment. For intracellular staining, cell surface stained cells were fixed and permeabilized using True‐nuclear™ transcription factor kit‐Biolegend (San Diego, CA) and stained for intracellular targets in accordance with the manufacturer's protocol. The data was obtained with BD FACSVerse and analyzed through BD FlowJo software (San Jose, CA).

### 4.7 Cytokine estimation

The cytokines were estimated in the culture SNs by sandwich ELISA. Briefly, 96‐well ELISA plates were coated with purified rat anti‐mouse IL‐6, IFN‐γ, IL‐17, IL‐12, TGF‐β, IL‐4, TNF‐α, IL‐2 (all at 2 μg/mL), and IL‐10 (4 μg/mL) antibodies in phosphate buffer (0.01 M‐pH 9.2 or pH 6) overnight (O/N) at 4°C as per the manufacturer's protocol BD Biosciences (San Diego, CA). The next day, blocking was done with BSA (1%) in PBS for 2 h at room temperature (RT). Subsequently, 50 μL samples (culture SNs) along with the respective standards of the recombinant cytokines were added to each well followed by O/N incubation at 4°C. After incubation and subsequent washing, 50 μL biotinylated anti‐mouse antibodies (2 μg/mL) for respective cytokines were added to plates and incubated for 2 h at RT. Further, plates were incubated with streptavidin‐HRP at 1:10,000 dilution at 37°C for 40 min. After subsequent washing, 1× TMB solution was used for color development and the reaction was stopped by H_2_SO_4_(7%). Absorbance was read at 450 nm on a spectrophotometer, Synergy H1, BioTek (Santa Clara, CA). The quantification of cytokines (pg/mL) was done using a standard curve of recombinant cytokines log2 serial dilutions.

### 4.8 Quantitative real‐time PCR (qRT‐PCR)

Trizol reagent was used to isolate total RNA from LPS‐stimulated DCs according to the manufacturer's protocol‐Invitrogen (Carlsbad, CA). RNA samples were incubated with amplification grade DNase1 enzyme to remove DNA contamination. 1 μg purified RNA with a standard ratio of 260/280 1.9–2.0 was used to synthesize cDNA with a reverse transcription kit, according to the manufacturer's protocol‐Applied Biosystems (Foster City, CA). Following that, qRT‐PCR was carried out using SYBR green PCR mix‐Applied Biosystems (San Diego, CA) according to the manufacturer's protocol. The real‐time PCR analysis was performed using the comparative Ct method on an Applied Biosystems step one plus PCR (Waltham, MA). The results are presented as a relative expression after normalization with β‐actin. The primer sequences used in PCR are listed in Table S1.

### 4.9 DC‐T cell co‐cultures

CD4^+^ T cells were purified from single‐cell suspension of the mouse splenocytes with BD IMag™ mouse CD4 T lymphocyte enrichment set–DM, BD Biosciences (San Diego, CA) by MACS negative selection following the manufacturer's protocol. Later, sorted T cells were co‐cultured with LPS‐stimulated DCs in a 1:5 ratio (DC: T cell) with a total seeding population of 2 × 10^5^ cells per well of 96‐well plates.

### 4.10 Proliferation assay

CD4^+^ T cells were labelled with carboxyfluorescein succinimidyl ester (CFSE, 2 μM)‐ Sigma Aldrich (St. Louis, MO) in PBS for 8 min at 37°C. Any unbound CFSE was washed with RPMI with 10% FCS. Stained cells were then co‐cultured with DCs in 96‐well flat bottom plates coated with anti‐mouse CD3 (5 μg/mL) and soluble anti‐mouse CD28 (2 μg/mL) antibodies at 37°C with 5% CO_2_ for 72 h. The proliferation of labeled T cells was assessed, using flow cytometry. The culture SNs were collected to later estimate the cytokines through ELISA. For syngeneic and allogeneic setup, CD4^+^ T cells were obtained from C57BL/6 and BALB/c mice, respectively.

### 4.11 In vitro Tregs induction assay

The same procedure was followed as described in DC‐T cell co‐culture procedure and maintained for up to 5 days for induction of T regulatory cells (Tregs). On the 5th day, cells were harvested and stained for Treg‐specific markers; anti‐mouse CD4‐FITC (# 11‐0042‐82), CD25‐PE‐Cy7 (# 25‐0251‐82), and FOXP3‐PE (# 12–5773‐82) all from eBioscience (San Diego, CA); and CD127‐APC (# 158205) Biolegend (San Diego, CA) fluorochrome‐tagged monoclonal antibodies.

### 4.12 Adoptive transfer of DCs

LPS‐stimulated DCs (5 × 10^6^) from different groups of mice viz. young, young dysbiotic, old and old‐LP were injected intravenously (i.v.) into old mice in 200 μL of PBS.

### 4.13 Nitric oxide (NO) production

After 24 hours, the culture SNs of LPS‐stimulated BMDCs were collected and NO was measured according to Griess method (Pahari et al., 2016). In brief, a 1:1 ratio of SNs was added to the Griess reagent (50 μL)‐ Sigma Aldrich (St. Louis, MO) and incubated for 5 min at room temperature. Later, the absorbance was measured at 550 nm. NO was quantified in comparison with sodium nitrite (NaNO_2_) as a standard (μM).

### 4.14 Phagocytosis assay

LPS‐stimulated DCs (1 × 10^6^ per sample) were incubated with dextran‐FITC (40,000 Da)‐ Sigma Aldrich (St. Louis, MO) at 37°C for 30 min, or on ice as a control, for the dextran antigen uptake. After proper washing, uptake was then assessed by flow cytometry. For phagocytic uptake of apoptotic bodies, Jurkat T cells labelled with CFSE were treated with actinomycin D for 14–15 h. to induce apoptosis in the same. Later, apoptotic cells were co‐cultured with LPS‐stimulated DCs for 3–4 h, and uptake was monitored by flow cytometry.

### 4.15 In vivo DC migration

LPS‐stimulated DCs from different groups of mice were labelled with CFSE (2 μM), and 5 × 10^6^ cells were adoptively transferred through an intravenous (i.v.) route into Old mice in 200 μL of sterile PBS. After 5 days, animals were sacrificed and cells from the spleen, mesenteric lymph node and Peyer's patches were stained for fluorochrome‐tagged anti‐mouse CD11c antibody. Later, the CD11c^+^CFSE^+^ population was monitored to assess migrated DC population. Furthermore, cells from the same tissues as mentioned were stained by fluorochrome‐tagged anti‐mouse CD4, CD127, CD25 and FOXP3 to assess the induction of Tregs using flow cytometry.

### 4.16 Inhibition of the NF‐κB pathway

The DCs harvested on the seventh day of culture were stimulated with LPS (1 μg/mL) in presence of a selective inhibitor for nuclear translocation of NF‐κB p65, JSH‐23 (7 μM) #481408, Sigma Aldrich (St. Louis, MO) in 24‐well plates (0.5 × 10^6^ cells/well) in 1 mL complete media for 24 h. Later, cells were washed with PBS after harvesting and used for further experiments.

### 4.17 Dextran‐FITC intestinal permeability assay

Evaluation of intestinal epithelial barrier permeability was done utilizing the dextran‐FITC permeability assay (Furuta et al., 2001; Meisel et al., 2018). In brief, mice were fasted for 4 h and then 60 mg dextran‐FITC (MW 4000), #46944, Sigma Aldrich (St. Louis, MO) per 100 g body weight was given through oral gavage. After 3 h of gavage, blood samples (400–500 μL) were obtained from the tail vein and plasma was extracted by centrifugation (2000 g for 10 min at 4°C). Thereafter, 50 μL blood plasma was transferred in duplicates into a flat‐bottom 96‐well plate (Corning, NY), and fluorescence reading was measured in fluorescence spectrophotometer setup in Synergy H1, BioTek (Santa Clara, CA) with emission and excitation wavelengths of 520 nm and 490 nm, respectively. Plasma dextran‐FITC concentration was assessed using a standard curve established by serial dilution of the same.

### 4.18 Western blotting

The whole cell lysate was prepared by lysing LPS‐stimulated DCs in RIPA lysis buffer (Tris HCl25 mM, NaCl150 mM, EDTA5 mM, Triton X‐1000.1%, sodium deoxycholate 1%, SDS 0.1%) supplemented with PMSF, phosphatase and protease inhibitor cocktail after 40 min incubation on ice. Lysates were centrifuged at 8000 *g* for 10 min, and the protein concentration of the lysate was measured by the BCA method. 40 μg of protein was added to Laemmli buffer and boiled (10 min, 95°C). Proteins were separated in 10–12% SDS‐PAGE gels and transferred to the PVDF membrane. Later, 5% BSA was used to block the membranes followed by incubation with the primary antibody on a shaker overnight at 4°C. All primary antibodies were diluted at a 1:500–1000 ratio for immunoblotting. HRP‐conjugated secondary anti‐mouse and anti‐rabbit antibodies were used (1:10000) for the detection of primary antibody binding. Each step included regular washings and incubations. Finally, blots were developed using Novex™ ECL Chemiluminescent Substrate Reagent Kit, Invitrogen‐Thermo Fisher Scientific, (Waltham, MA) and visualization was done on iBright™ FL1500 Imaging System, Invitrogen‐Thermo Fisher Scientific (Waltham, MA). Blot analysis and quantification were done using ImageJ analysis software. Abs to phospho p65, p65, anti‐ IDO, iNOS, arginase1, and β‐actin (loading control) were used for Western blotting.

### 4.19 Glucose uptake assay

ACK lysed bone marrow cells (BMCs) and LPS‐stimulated DCs were incubated with 10 μM of 2‐NBDG (2‐(N‐(7‐Nitrobenz‐2‐oxa‐1,3‐diazol‐4‐yl) Amino)‐2‐Deoxyglucose) in glucose‐free RPMI‐1640 + 10% FCS at 37°C/5% CO_2_ for 30 min. Thereafter, cells were washed and 2‐NBDG uptake was monitored through flow cytometry, BD FACSVerse™ (San Jose, CA) and data were analysed through BD FlowJo software (San Jose, CA).

### 4.20 Fecal DNA extraction

Aseptically obtained mice fecal samples were immediately placed on ice and kept at −80°C for later use. Zymo Research Fecal DNA Isolation Kit (Irvine, CA) was used to isolate fecal DNA from 100 mg of fecal pellets, according to the manufacturer's procedure. Quantification was done on a NanoDrop spectrophotometer, SYNERGY H1‐BioTek (Santa Clara, CA). The quantified DNA was then directly used for real‐time PCR analysis. 50 ng of extracted genomic DNA from fecal samples was used as a template for a real‐time PCR reaction with genus/species‐specific primers (0.2 μM), and SYBR green PCR Master‐mix, Applied Biosystems (San Diego, CA). Each cycle of PCR reaction was 10 min at 95°C, then 40 cycles of 30 s at 95°C, 1 min at 60°C and performed in step one plus PCR, Applied Biosystems (Waltham, MA). Data were represented as relative abundance for each genus/species after normalization with bacterial universal primer/DNA as an internal control.

### 4.21 16S rRNA sequencing for gut microbiota analysis

DNA was isolated as per the already discussed protocol from mice fecal samples. Later, DNA was further processed for amplicon‐based sequencing of the V3‐V4 hyper‐variable region of the 16S rRNA gene. Fusion primer 16S amplicon PCR primers Fw 5′ TCGTCGGCAGCGTCAGATGTGTATAAGAGACAGCCTACGGGNGGCWGCAG 3′ and Rv 5′ GTCTCGTGGGCTCGGAGATGTGTATAAGAGACAGGACTACHVGGGTATCTAATCC 3′ (Klindworth et al., 2013) was used to amplify V3‐V4 region of 16S rRNA using 12.5 ng of DNA. Sequencing libraries were prepared from amplified V3‐V4 region of 16S rRNA by Index PCR using Nextera XT index kit as per manufacturer instructions (Illumina, #15044223 Rev. B). The quality of the final libraries was checked using high‐sensitivity D1000 screen tape in Tape‐Station 2200 Agilent Technologies (Santa Clara, CA), and final library quantification was performed in Qubit Fluorometer. Paired‐end (2 × 250 bp) sequencing of these libraries was performed in NovaSeq 6000 (Illumina, San Diego, CA). Sample preparation and sequencing were done at the National Institute of Biomedical Genomics (Kalyani, India). The raw data were first checked for quality, and then, DADA2 was used to remove chimeric information. The demultiplexed sequences were then trimmed to lengths of 250 and 230 for forward and reverse, respectively. After that, the frequency table and data were obtained. Model classifier, which was created using the reference Green gene database, was used to classify taxa. The taxonomic plots were then created using the model classifier parameters such as forward primer CCTACGGGNGGCWGCAG and reverse primer GACTACHVGGGTATCTAATCC. The OTU table has been created from taxonomic classification. MAFFT was used to align the sequences, which were then masked to obtain masked aligned sequences. Subsequently, for diversity analysis, phylogenetic trees were created. With the parameter sampling depth 582,778, PCoA and diversity analysis plots were obtained using rooted and unrooted trees.

### 4.22 Microarray‐based gene expression analysis

RNA was isolated from DCs after LPS stimulation (1 μg/mL) using TRIzol reagent according to the manufacturer's protocol, Sigma Aldrich (St. Louis, MO). Later, the DNase1 enzyme was added for the digestion and removal of genomic DNA. Thereafter, RNA was quantified with the NanoDrop ND‐100 Spectrophotometer, NanoDrop Technologies (Wilmington, DE), and quality for the same was examined with the Tapestation 4200™, Agilent Technologies (Santa Clara, CA). Samples with RIN (RNA integrity number) score of more than 6 and a 28S:18S rRNA ratio of around 2∶1 were included for the further process. Thereafter, labelling and hybridization were done using Agilent one‐color (Cy3 fluorochrome), a microarray‐related gene expression platform in accordance with the manufacturer's instructions for evaluating mRNA expression. Briefly, 500 ng of RNA was labelled with a one‐color Low‐Input Quick Amp labelling kit, 5190–2305 (Agilent Technologies, Palo Alto, CA, USA). Cy3‐labelled mRNA samples were hybridized onto a mouse 8 × 60 K Gene Expression V2 Array kit, G4858A, Agilent Technologies, (Palo Alto, CA) for 16 h at 55°C in a rotator oven with subsequent washing. DNA microarray scanner, Agilent Technologies, (Palo Alto, CA) was used to scan the array slides, and Agilent Feature Extraction software 10.5 (Palo Alto, CA) to extract hybridization signals. Thereafter, with the help of limma R package, the quantile normalization method was implemented to normalize the quantitative microarray data. Using the same package, differential feature estimation was performed by lmFit () function to fit a linear model to expression data of each feature and eBayes (empirical Bayes) function used to obtain adj.*p*.value and logFC. Threshold adj.*p*.value ≤ 0.05 and abs (logFC) ≥ 0.5 were used to obtain significant differential features. R package gprofiler2 was used for the enrichment of functional annotations. Summarization of gene ontology and treemap plot were done with R package rrvgo.

#### Statistical analysis

One‐way ANOVA followed by Tukey's multiple comparison test and two‐way ANOVA followed by Sidak's multiple comparisons test were done using GraphPad Prism 6 software (San Diego, CA). A *p* value <0.05 was considered significant.

## AUTHOR CONTRIBUTIONS

HB performed the experiments and contributed to experimental design, data analysis, discussion and writing. RPS helped in flow cytometry data acquisition with HB and analyzed the 16S rRNA sequencing data. SS conducted experiments with HB. JNA conceptualized, designed and supervised the study, and contributed to the discussion and writing of the manuscript. RK conceptualized, designed and supervised the study, analyzed the data and wrote the manuscript. All authors revised and commented on the manuscript. All authors approved the final manuscript.

## FUNDING INFORMATION

This study is supported by the grants from CSIR, India grants OLP134, HUM (No. BSC0119) and CSIR‐FIRST (No. MLP062).

## CONFLICT OF INTEREST STATEMENT

The authors declare that they have no conflict of interest.

## Supporting information


Figure S1

Figure S2

Figure S3

Figure S4

Figure S5

Table S1

## Data Availability

The 16 s rRNA sequencing data from this study have been deposited at the GenBank Sequence Read Archive with the accession number PRJNA879291. The microarray raw data are submitted to the GEO repository with GEO accession number GSE 213155.
