# Supplementary material for: Age‐mediated gut microbiota dysbiosis promotes the loss of dendritic cells tolerance
Source: Aging Cell. 2023 May 9;22(6):e13838. doi: 10.1111/acel.13838 (PMC10265174; doi:10.1111/acel.13838)
Supplement: Supplementary file 1 — Figure S1 [file ACEL-22-e13838-s004.pdf]

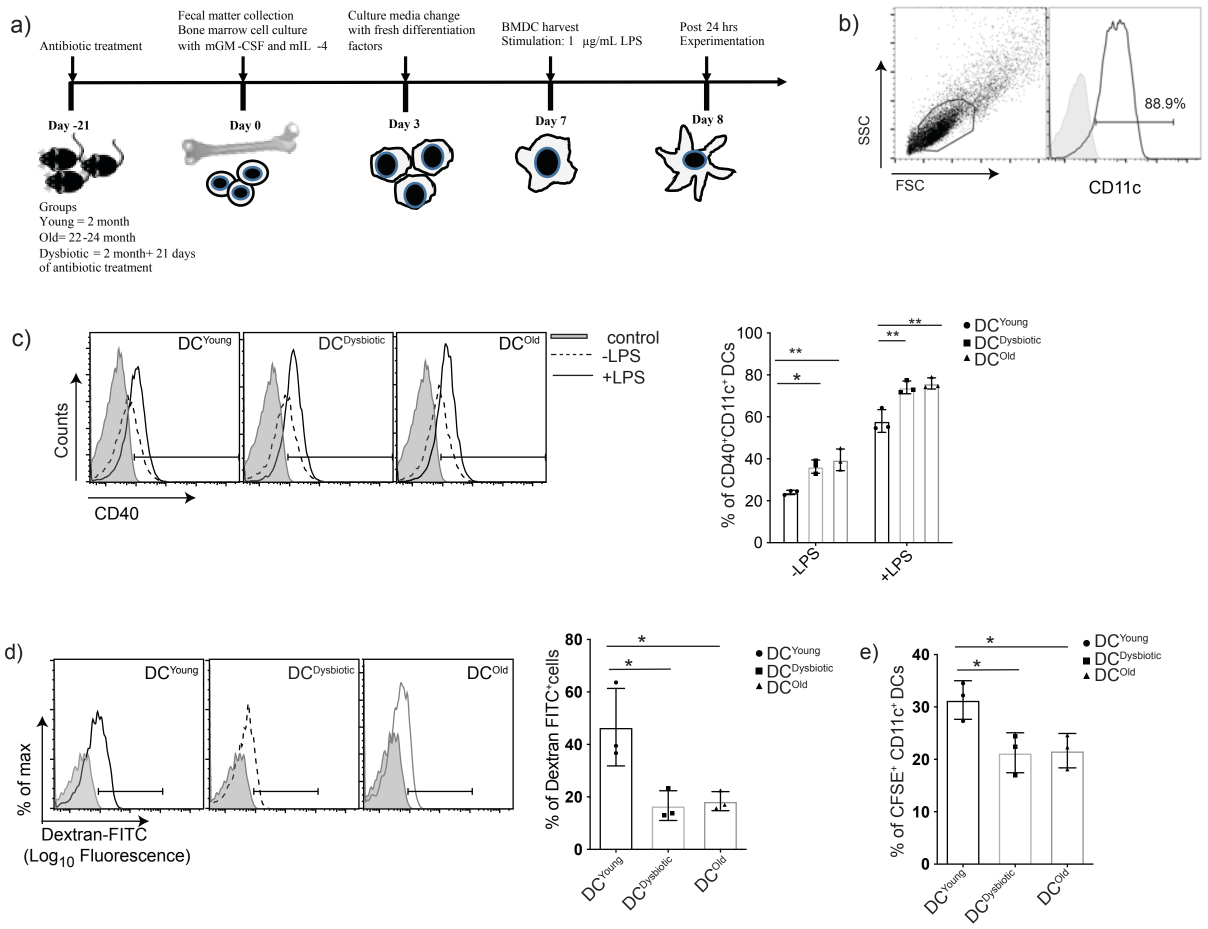

Figure S1. Altered DCs phenotype and impaired phagocytic activity is induced by aging or antibiotic treatment of young mice due to gut dysbiosis.

a) Experimental strategy: Young mice were administered an antibiotic cocktail for 21 days. Later, fecal pellets were collected from young control, young dysbiotic and old group. BMCs were cultured for seven days followed by LPS stimulation for 24 hours (1µg/ml); b) Histogram depicts the gating strategy of $CD11c^{+}$DCs and purity; c) Representative histogram and frequency of $CD11c^{+}CD40^{+}$ cells before and after LPS stimulation; d) Frequency of dextran-FITC uptake, assessed by flow cytometry and e) Flow cytometry data showing uptake of CFSE labeled apoptotic Jurkat cells by DCs. The data (mean±SD) are from three independent experiments, with each point representing a pool of three animals for one independent experiment, n=3 mice/group. Statistical analysis was done by One-way ANOVA and Tukey's multiple comparison test except (Fig S1 c), where Two-way ANOVA and Sidak's multiple comparisons test was performed. $*p < 0.05$, $**p < 0.01$.
