# Supplementary material for: Age‐mediated gut microbiota dysbiosis promotes the loss of dendritic cells tolerance
Source: Aging Cell. 2023 May 9;22(6):e13838. doi: 10.1111/acel.13838 (PMC10265174; doi:10.1111/acel.13838)
Supplement: Supplementary file 2 — Figure S2 [file ACEL-22-e13838-s003.pdf]

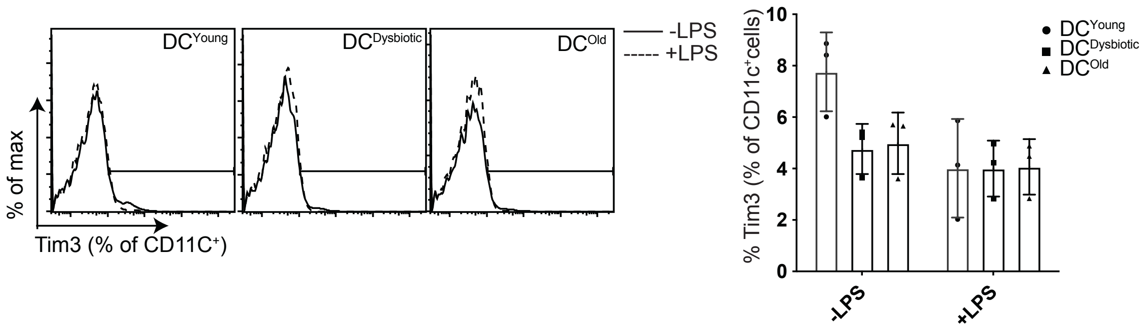


**Figure S2. Aging or antibiotic treatment induced gut dysbiosis alters co-inhibitory phenotype of DCs**

Representative histogram and frequency of $CD11c^{+}TIM3^{+}$ cells before and after LPS stimulation. Data (mean±SD) are of three independent experiments, with each point representing a pool of three animals for one independent experiment, n=3 mice/group. Two-way ANOVA and Sidak's multiple comparisons test were performed for statistical analysis. ns: non-significant.
