# Supplementary material for: Age‐mediated gut microbiota dysbiosis promotes the loss of dendritic cells tolerance
Source: Aging Cell. 2023 May 9;22(6):e13838. doi: 10.1111/acel.13838 (PMC10265174; doi:10.1111/acel.13838)
Supplement: Supplementary file 3 — Figure S3 [file ACEL-22-e13838-s001.pdf]

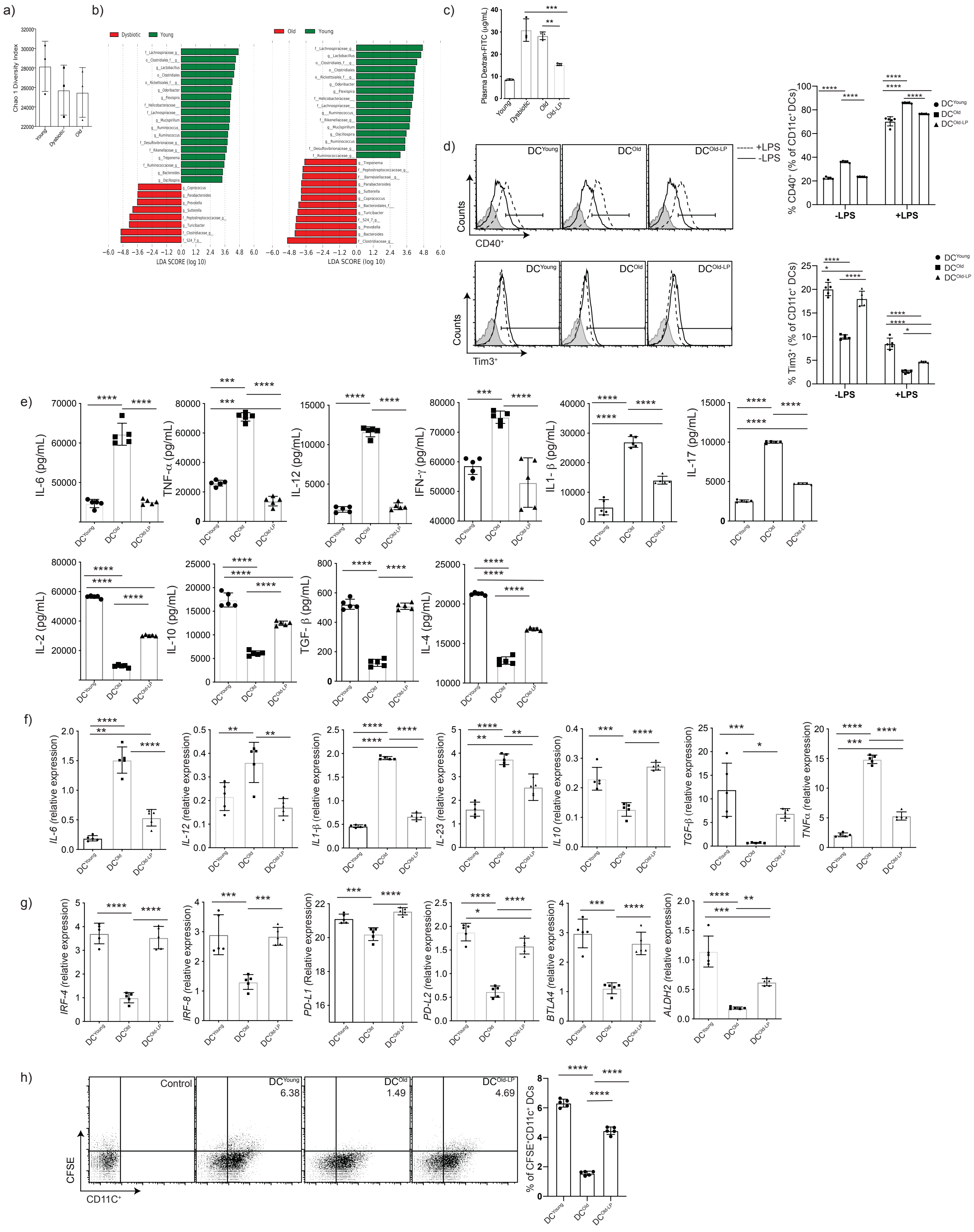

Figure S3. Alterations of the gut microbiota with aging and antibiotics exposure with Immunomodulatory role of *L. plantarum* in old DCs.

After 21 days of antibiotic treatment of young mice, DNA was isolated from the feces (100 mg) of young, young dysbiotic, and old mice for 16S rRNA sequencing. a) Chao1 index depicting alpha diversity; b) LEfSe plots representing a significant difference in the relative abundance at genus level between groups; c) Animals were fasted for 4 hours and later gavaged with dextran-FITC (60 mg/100g body weight). Dextran-FITC permeabilized through gut epithelia was estimated in the blood plasma as depicted by a bar graph representing plasma concentration of dextran-FITC (μg/ml); After 21 days of LP replenishment in old mice (Old-LP), BMDCs culture was set up for 7 days for later experimentation in DC^{Young}, DC^{Old} and $DC^{OLD\text{-}LP}$. d) Histogram and bar graph depicting percentage population of $CD40^+$ and $TIM3^+$ DCs gated on $CD11c^+$ cells; e) ELISA-based estimated levels (pg/mL) of secreted pro-inflammatory IL-6, TNF-α, IL-12, IFN-γ, IL-1β, IL-17 and anti-inflammatory cytokines TGF- β, IL-4, IL-2, IL-10 in DC-T cell co-culture SN after 72 hours; f) RT-PCR data for relative expression of inflammatory *Il-6*, *Il-12*, *Tnfa*, *Il-23*, *Il1b*, and anti-inflammatory cytokine genes *Tgfb*, *Il-10*; g) relative expression of the tolerance associated genes *Irf4*, *Irf8*, *Pdl1*, *Pdl2*, *Btla4* and *Aldh2*, and h) Representative dot plot and frequency of CFSE-labelled apoptotic cells uptake by DCs as assessed by flow cytometry. Data (mean±SD) are of 5 animals, with each point in the bar graph indicating one animal, (n=5/group) except (Fig S3 a and b), which is of three independent experiments, with each point representing a pool of three animals for one independent experiment, (n=3 mice/group) and (Fig S3 c), with each point in the bar graph indicating one animal (n=3/group). Statistical analysis was done by One-way ANOVA and Tukey's multiple comparison tests except for (Fig S3 d), where Two-way ANOVA and Sidak's multiple comparisons test were performed. *$p < 0.05$, **$p < 0.01$, ***$p < 0.001$, ****$p < 0.0001$.
