# Supplementary material for: Age‐mediated gut microbiota dysbiosis promotes the loss of dendritic cells tolerance
Source: Aging Cell. 2023 May 9;22(6):e13838. doi: 10.1111/acel.13838 (PMC10265174; doi:10.1111/acel.13838)
Supplement: Supplementary file 4 — Figure S4 [file ACEL-22-e13838-s002.pdf]

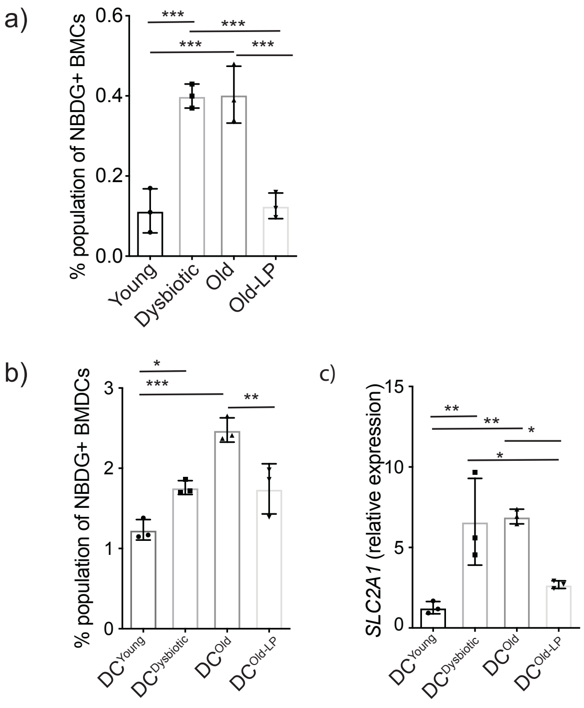

Figure S4. Replenishment of the gut of old mice with *L. plantarum* reinstates the metabolic function of old DCs.

BMCs and LPS-stimulated DCs were incubated with 10µM of 2-NBDG for 30 mins. After that, cells were washed, and 2-NBDG uptake was monitored through flow cytometry. a) Frequency (%) of $NBDG^{+}$BMCs; b) Frequency (%) of $NBDG^{+}$BMDCs c) RT-PCR data for relative expression of *Slc2a1* gene encoding for GLUT1 glucose transporter. Data (mean±SD) is of three animals, with each point in the bar graph representing one animal (n=3/group). Statistical analysis was done by One-way ANOVA and Tukey's multiple comparison test.
