# Supplementary material for: Age‐mediated gut microbiota dysbiosis promotes the loss of dendritic cells tolerance
Source: Aging Cell. 2023 May 9;22(6):e13838. doi: 10.1111/acel.13838 (PMC10265174; doi:10.1111/acel.13838)
Supplement: Supplementary file 5 — Figure S5 [file ACEL-22-e13838-s006.pdf]

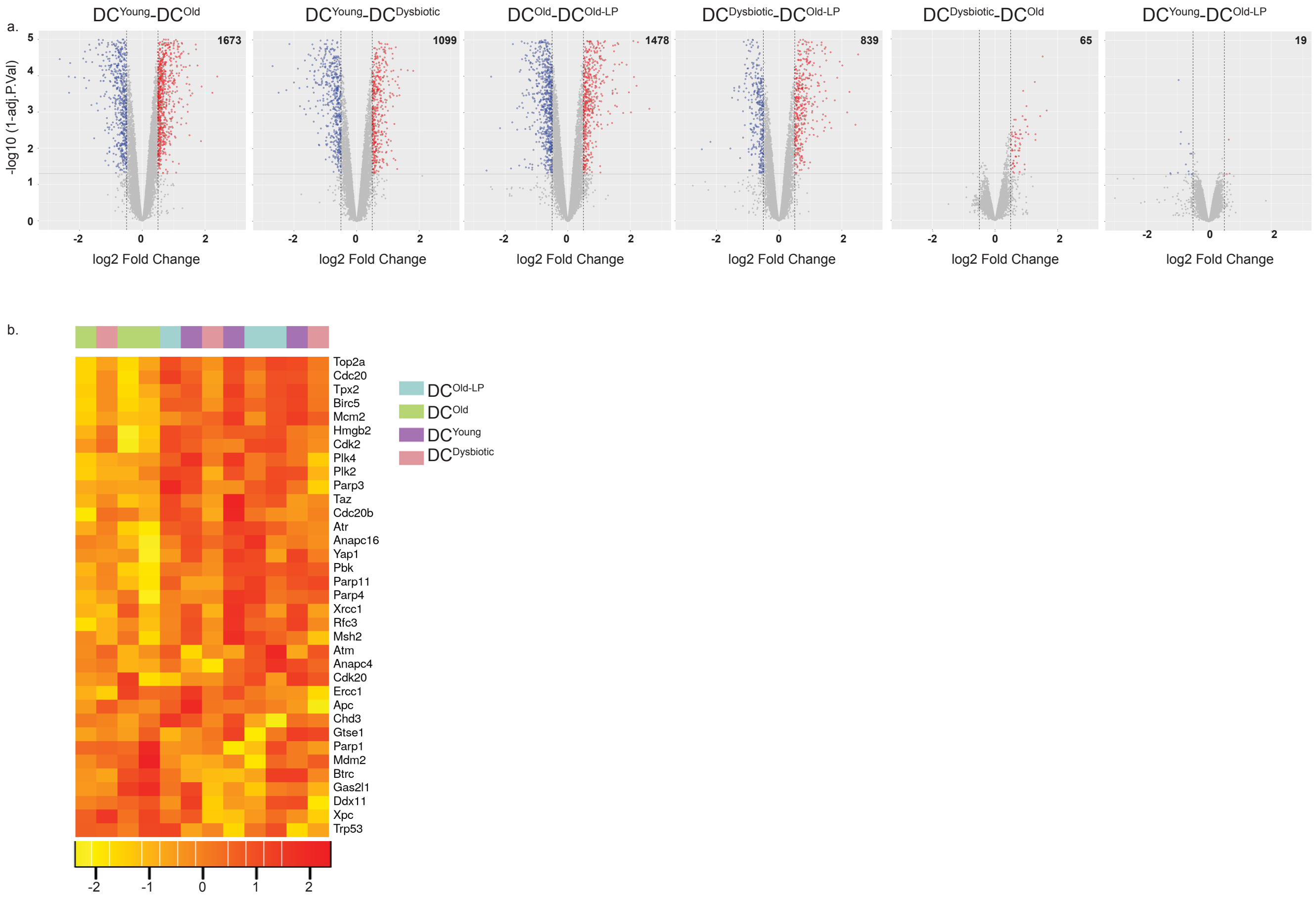

Figure S5. Differential gene expression summary.

a) Volcano plots representing the log2(Fold Change) and -log10(P value) distribution of differentially expressed genes as a scatter plot from various comparisons; b) heatmap representing the differentially expressed features associated with cell cycle and DNA repair. Data are from three independent sets, with each data point representing a pool of three animals (n=3 mice/group).
