# Supplementary material for: Age‐mediated gut microbiota dysbiosis promotes the loss of dendritic cells tolerance
Source: Aging Cell. 2023 May 9;22(6):e13838. doi: 10.1111/acel.13838 (PMC10265174; doi:10.1111/acel.13838)
Supplement: Supplementary file 6 — Table S1 [file ACEL-22-e13838-s005.pdf]

Supplementary Table 1

Mouse qRT-PCR primer sequences:

Gene	Sequence
Tnfα	Fw: 5′-CATCTTCTCAAAATTCGAGTGACAA-3′<br>Rv: 5′-TGGGAGTAGACAAGGTACAACCC-3′
Il-10	Fw: 5′-GGTTGCCAAGCCTTATCGGA-3′<br>Rv: 5′-ACCTGCTCCACTGCCTTTGCT-3′
Il-12	Fw: 5′-GGAAGCACGGCAGCAGCAGAATA-3′<br>Rv: 5′-AACTTGAGGGAGAAGTAGGAATGG-3′
Il-6	Fw: 5′-GAGGATACCACTCCCAACAGACC-3′<br>Rv: 5′-AAGTGCATCATCATCGTTGTTCATACA-3′
Il-23	FW: 5′-TGTGCCCCGTATCCAGTGT-3′<br>Rv: 5′-CGGATCCTTTGCAAGCAGAA-3′
Il1b	Fw: 5′-CAACCAACAAGTGATATTCTCCATG-3′<br>Rv: 5′-GATCCACACTCTCCAGCTGCA-3′
Tgfb	Fw: 5′-TAAAGAGGTCACCCGCGTGCTAAT-3′<br>Rv: 5′-ACTGCTTCCCGAATGTCTGACGTA-3′
Irf4	Fw: 5′-GCCCAACAAGCTAGAAAG-3′<br>Rv: 5′-TCTCTGAGGGTCTGGAAACT-3′
Irf8	Fw: 5′-GAGCGAAGTTCCTGAGATGG-3′<br>Rv: 5′-TGGGCTCCTCTTGGTCATAC-3′
Aldh2	Fw: 5′-GCTGGGCTGACAAGTACCAT-3′<br>Rv: 5′-TTGATCAAGTTGGCCACGTA-3′
Btla4	Fw: 5′-GCAGGACTTGGCTGCTTTAC-3′<br>Rv: 5′-AGTTCCTGATGGCAGTGCTT-3′
Pdl1	Fw: 5′-TGCGGACTACAAGCGAATCACG-3′<br>Rv: 5′-CTCAGCTTCTGGATAACCCTCG-3′
Ido	Fw: 5′-AGTTGGGCCTGCCTCC TATTC-3′<br>Rv: 5′-TGTCGTGCAGTGCCTTTTCCAAT-3′
iNOS	Fw: 5′-CGAAACGCTTCACTTCCAA-3′<br>Rv: 5′-TGAGCCTATATTGCTGTGGCT-3′

Arginase1	Fw: 5′-TTTTTCCAGCAGACCAGCTT-3′<br>Rv: 5′-AGAGATTATCGGAGCGCCTT-3′
RelA	Fw: 5′-GACCAACAATAACCCCTTTCAC-3′<br>Rv: 5′-GTTTGAGATCTGCCCTGATGG-3′
Pdl2	Fw: 5′-CATCGCTTTGATCTTCCTGG-3′<br>Rv: 5′-CCTGAAAGTCATTAGGAGCC-3′
β-actin	Fw: 5′-AGAGGGAAATCGTGCGTGAC-3′<br>Rv: 5′-CAATAGTGATGACCTGGCCGT-3′
Lactobacillus	Fw: 5′-AGCAGTAGGGAATCTTCCA-3′<br>Rv: 5′-CACCGCTACACATGGAG-3′
L. plantarum	Fw: 5′-TGGATCACCTCCTTTCTAAGGAAT-3′<br>Rv: 5′-TGTTCTCGGTTTCATTATGAAAAAATA-3′
All bacteria (Universal primer)	Fw: 5′-ACTCCTACGGGAGGCAGCAGT-3′<br>Rv: 5′-ATTACCGCGGCTGCTGGC-3′
